# Effectiveness of facility-based personalized maternal nutrition counseling in improving child growth and morbidity up to 18 months: A cluster-randomized controlled trial in rural Burkina Faso

**DOI:** 10.1371/journal.pone.0177839

**Published:** 2017-05-25

**Authors:** Laetitia Nikièma, Lieven Huybregts, Yves Martin-Prevel, Philippe Donnen, Hermann Lanou, Joep Grosemans, Priscilla Offoh, Michèle Dramaix-Wilmet, Blaise Sondo, Dominique Roberfroid, Patrick Kolsteren

**Affiliations:** 1 Institut de Recherche en Sciences de la Santé (IRSS), Ministry of Scientific Research and Innovation, Ouagadougou, Burkina Faso; 2 Poverty, Health and Nutrition Division, International Food Policy Research Institute, Washington DC, United States of America; 3 Research Unit 204 ‘Nutripass’, Institut de Recherche pour le Développement, Montpellier, France; 4 Ecole de Santé Publique, Centre de Recherche ‘Politiques et Systèmes de Santé—Santé Internationale’, Université Libre de Bruxelles (ULB), Campus Erasme, Brussels, Belgium; 5 PXL University College, Healthcare Department, Guffenslaan, Hasselt, Belgium; 6 Push Against Malaria Health Initiative (PAMHI), Department of Software Management and Programming in PAMHI, Okota Lagos, Nigeria; 7 Department of Public Health, Child Health and Nutrition Unit, Institute of Tropical Medicine, Antwerp, Belgium; 8 Department of Food Safety and Food Quality, Ghent University, Ghent, Belgium; TNO, NETHERLANDS

## Abstract

The period from conception to 24 months of age is a crucial window for nutrition interventions. Personalized maternal counseling may improve childbirth outcomes, growth, and health. We assessed the effectiveness of facility-based personalized maternal nutrition counseling (from pregnancy to 18 months after birth) in improving child growth and health in rural Burkina Faso. We conducted a paired cluster randomized controlled trial in a rural district of Burkina Faso with 12 primary health centers (clusters). Healthcare providers in the intervention centers received patient-centered communication and nutrition counseling training. Pregnant women in the third trimester living in the center catchment areas and intending to stay for the next 2 years were prospectively included. We followed 2253 mother-child pairs quarterly until the child was aged 18 months. Women were interviewed about counseling experiences, dietary practices during pregnancy, and their child’s feeding practices and morbidity history. Anthropometric measurements were taken at each visit using standardized methods. The primary outcomes were the cumulative incidence of wasting, and changes in child weight-for-height z-score (WHZ). Secondary outcomes were the women’s prenatal dietary practices, early breastfeeding practices, exclusive breastfeeding, timely introduction of complementary food, child’s feeding frequency and dietary diversity, children’s mean birth weight, endpoint prevalence of stunting, and cumulative incidence of diarrhea, fever, and acute respiratory infection. All analyses were by intention-to-treat using mixed effects models. The intervention and control arms each included six health centers. Mothers in the intervention arm had a significantly higher exposure to counseling with 11.2% for breastfeeding techniques to 75.7% for counseling on exclusive breastfeeding. Mothers of infants below 6 months of age in the intervention arm were more likely to exclusively breastfeed (54.3% *vs* 42.3%; Difference of Proportion (DP) 12.8%; 95% CI: 2.1, 23.6; *p* = 0.020) as compared to the control arm. Between 6 and 18 months of age, more children in the intervention arm benefited from the required feeding frequency (68.8% *vs* 53.4%; DP 14.1%; 95% CI: 9.0, 19.2; *p*<0.001) and a larger proportion had a minimum dietary diversity (28.6% *vs* 22.0%; DP 5.9%; 95% CI: 2.7, 9.2; *p*<0.001). Birth weight of newborns in the intervention arm was on average 84.8 g (*p* = 0.037) larger compared to the control arm. However, we found no significant differences in child anthropometry or morbidity between study arms. Facility-based personalized maternal nutrition counseling was associated with an improved prenatal dietary practices, Infant and Young Child Feeding practices, and child birth weight. Complementary strategies are warranted to obtain meaningful impact on child growth and morbidity. This includes strategies to ensure good coverage of facility-based services and effective nutrition/care practices in early childhood.

## Introduction

Inappropriate care and feeding practices during the first 2 years of life increase the burden of child undernutrition and contribute to childhood morbidity and mortality worldwide, and particularly in sub-Saharan Africa [1, 2]. Common inappropriate complementary feeding practices include lack of exclusive breastfeeding during the first 6 months of life, untimely introduction of complementary food, poor diversity of children’s diets, and low feeding frequencies [3, 4]. Inappropriate feeding practices may be related to insufficiencies in food availability and variety. However, caregivers’ cultural beliefs, lack of knowledge of optimal feeding practices, inappropriate feeding behavior, and lack of awareness about the amount needed to feed a child, feeding frequency, and food types are modifiable factors that can contribute to the deterioration of a child’s nutritional status [5, 6]. Therefore, interventions aimed to counsel mothers on appropriate Infant and Young Child Feeding (IYCF) practices are needed to improve child health and growth, especially in rural areas.

Changes in behavior related to feeding practices can be achieved through well-designed and well-implemented behavior change communication interventions. During pregnancy, several nutritional interventions to improve maternal and child undernutrition have been assessed, including prenatal dietary counseling. Reviews of previous studies indicate that antenatal nutrition counseling on increasing energy and protein intake during pregnancy is effective in reducing the risk of preterm birth. However, the effects on child birth weight and the long-term health of the baby are uncertain [7, 8]. After birth, counseling has been shown to increase caregivers’ knowledge and improve breastfeeding, complementary feeding, and growth in young children [9–11].

The choice of appropriate platforms to deliver nutrition education interventions is crucial to achieve good coverage and to ensure the quality of the intervention. The use of lay healthcare workers to deliver preventive maternal and child interventions is a promising strategy [12], although most recent systematic reviews note that current evidence is insufficient [13–15]. Many factors interact to influence lay healthcare workers’ performance in low- and middle-income countries, including health system policy and practices in health centers [16]. Given resource constraints, low-income countries often depend on health facilities as the primary platform to deliver maternal and child health and nutrition services. The first 1000 days of life, from conception to 2 years of age is a crucial window of opportunity during which nutrition-specific interventions are recommended and have lasting benefits [17–22]. This window coincides with regular prenatal and postnatal visits to health facilities for promotional, preventive, and curative services. In 2010, about 88% of pregnant mothers globally had at least one antenatal visit, and 50% had a postnatal visit to a healthcare facility [23]. These contacts represent opportunities to provide counseling on nutrition and health practices during pregnancy and infancy through healthcare worker–caregiver interaction.

In most Sub-Saharan countries, there are many missed opportunities to deliver nutrition services during routine prenatal, postnatal, and child care consultations because healthcare providers often lack essential communication skills, resulting in inefficient nutrition counseling [24, 25]. Nutrition counseling training may change healthcare providers’ behavior and improve caregiver knowledge acquisition and practices [26–28]. Patient-centered communication improves the relationship between health provider and patient. This approach provides additional opportunities to consider the patient’s situation and provide treatment and advice tailored to their needs and socioeconomic status [29]. Training healthcare providers on patient-centered communication positively influences treatment compliance, behavior change, and follow-up appointments in other health areas [30, 31]. However, there is uncertainty about how routine contacts can be effectively used to promote appropriate feeding and child growth. The nutrition and health impact of preventive nutrition and health interventions delivered by primary healthcare staff merits evaluation through rigorous studies.

This study aimed to assess the effectiveness of training primary healthcre providers to deliver patient-centered, facility-based nutrition counseling from pregnancy to 18 months after birth to improve prenatal and postnatal dietary practices, and to support child further growth and health.

## Materials and methods

### Setting

This study was conducted in the health district of Houndé, located 250 km west of Ouagadougou, Burkina Faso’s capital city. The location was chosen as we had previously carried out two randomized controlled trials in this district [32, 33] and our collaboration with the health services and population was effective. Houndé has a population of 294,865 inhabitants, one general district hospital, and 27 government primary health centers.

At the time of the present study, each primary health center had at least three primary healthcare providers. Each primary health center offers a minimum package of preventive, promotional, and curative services for women and children. Preventive services include immunization (free for children under 5 years of age), deworming, and vitamin A supplementation. Promotional services include antenatal care, the Child Growth Monitoring and Promotion Program (GMP), and group communication on behavior change organized at the health center level. Such group communication may covered appropriate breastfeeding and complementary feeding practices, feeding of sick children, promotion of GMP session attendance, immunization promotion, insecticide-treated mosquito net use, common disease management (diarrhea, malaria, acute respiratory infection), antenatal care attendance, folic acid and iron supplementation during pregnancy, intermittent preventive treatment of malaria in pregnancy, pregnant women’s dietary needs, family planning, and hygiene and sanitation. Curative services include sick children consultations following the Integrated Management of Child Illness strategy. These services are continuous with a referral system to the general hospital. The average catchment area for primary health facilities had an average radius of 8.8 km in 2014, with 63% of the population living within 5 km of a facility [34]. In 2014, the average number of contacts with health centers was 1.8 per year for children under 5 years old in the district [34]. The health district has one pediatrician trained in nutrition responsible for monitoring all district nutrition activities. Treatment of acute malnutrition includes community-level passive case detection using mid-upper arm circumference measurements, referral to health centers for confirmation, and outpatient treatment of uncomplicated cases of severe acute malnourished children using ready-to-use therapeutic foods. Cases with medical complications or lack of appetite are hospitalized at the district hospital. Houndé had no specific services for moderate acute malnutrition cases at the time of this study (2008). Less than 6% of households in the region where the district is located are food insecure, against 19% nationally [35]. A situational analysis was conducted in the district in 2008, before implementation of the present intervention. This situational analysis comprised an in-depth investigation of all 27 primary health facilities to assess the functioning of maternal and child health services (observation of prenatal care, GMP sessions, and sick children consultations). After this detailed analysis, 12 health centers were selected as sites for the trial. The number of sites was limited by budget and logistical constraints. Selection was based on the availability of human resources able to be involved in the intervention delivery, and the availability of the minimum package of maternal and child services recommended nationally for first-line health facilities.

A survey in a representative sample of 3968 children was conducted in the catchment areas of the 12 selected health centers to collect data on population characteristics, including the prevalence of stunting and wasting, feeding practices, and morbidity rates and their determinants in children under 5 years old. From this pre-study we observed that the average household size was 8±5 (mean±SD), with 2±1 (mean±SD) children younger than 2 years. Most women (86.6%) never attended school. The principle economic activity in the district was agriculture (96%). Nearly 32% of households used an unprotected well or pond as a source of water for consumption. Most households (67.8%) had no toilet or latrine and people often defecated in the open. In terms of breastfeeding and complementary feeding practices, 51.4% of children aged <6 months did not receive colostrum at birth, and the average duration of breastfeeding was 15.9±5.1 months. Complementary food was introduced before 6 months in 39% of children, with the principal complementary food being simple cereal porridge (85.2%). The mean dietary diversity score based on nine food groups was 2.0±1.2 for children aged 6–59 months. Furthermore, we found that 67.6% of pregnant women attending antenatal consultations did not receive prenatal dietary intake advice. In addition, 75.8% of caregivers attending healthy child consultations did not receive advice on complementary feeding. The pilot study also found that 16.4% of children under 5 years of age were wasted, 38.7% were stunted, and the overall morbidity rate was 38.7% (S1 Table). The average birth weight was 2,926±502 g, and the low birth weight prevalence was 14.1%.

### Study design

We conducted a cluster randomized trial to assess the effect of preventive counseling during pregnancy and childhood on childbirth outcomes, child nutrition status, and morbidity. Healthcare workers were trained to offer counseling during their usual contacts with women. In the Burkina Faso health system, there are a limited number of healthcare providers in health facilities who interact with users daily. In addition, women attending the same health center usually live in the same community, and may communicate with each other and exchange experiences from that health center. Therefore, a cluster randomized design was the most appropriate design for the present study.

A cluster was defined as the catchment area of a primary health center. All primary health centers that had functioned for at least 1 year at the time of the study were eligible (27 health centers in total). From these 27 health centers, 12 were selected to participate in this study. Selection was based on the availability of human resources in the health centers (at least three primary healthcare providers, as recommended by national policy), the actual existence and regularity of delivery of the minimum package of activities recommended at a national level for first-level health facilities (prenatal visits, healthy child consultations, immunization sessions, sick children consultations), and health center performance based on the number of children under 24 months who had attended a healthy child consultation the previous month and the rate of measles immunization in 2007 (S2 Table). As this research project was based on healthcare provider participation, the last criterion was healthcare providers’ willingness to be involved in a formative research project. This was assessed during group discussion with healthcare providers during an initial visit to each health center to present the research project.

The 12 clusters were pair-matched using three criteria: i) distance to the district hospital; ii) mean household socioeconomic scores; and iii) child morbidity rate and prevalence of wasting and stunting. Data for the pair-matching was obtained from the 2008 pilot survey (S2 Table). In each pair, one health center was randomly allocated to the intervention arm, and the other to the control arm. Randomization was conducted publicly. For each pair of health centers, two identical pieces of paper were numbered corresponding to each health center and put into a basket. A volunteer not involved in the study was asked to choose a paper for the intervention center. After the first choice for the intervention center, the second center in that pair was systematically allocated to the control arm. No allocation concealment and blinding were possible. All participating centers, staff, and mothers were aware of the study arm to which they were assigned. Consent was obtained from each health center management committee, and a committee representative took part in the randomization session. No changes occurred in the trial conduct or outcomes after the study began.

### Population and sample size

The study population comprised pregnant women and their offspring living in the catchment areas of the 12 selected health centers for at least 6 months, and not planning to leave in the next 2 years.

The primary outcome used to compare the two groups was the incidence of wasted children. As no data were available on the incidence of acute malnutrition in the study area before the intervention, the prevalence of global acute malnutrition (16.4%) found in children under 5 years old in the 2008 pilot study was used as a proxy. Sample size calculation was based on the hypothesis that the intervention would contribute to a reduction of 5% in the prevalence of wasting in children under 5 years old in the area. The coefficient of variation between clusters within the matched pairs was conservatively estimated at 0.25.We used the formula proposed by Hayes et al. for a pair-matched cluster design to calculate the required sample size [36]. Taking into account an estimated 20% of incomplete or missing observations, this calculation resulted in a sample size of 180 children per cluster or 1,080 subjects per study arm, using 80% power and 95% confidence interval (CI).

To evaluate the intervention effect, a cohort of pregnant women in their third trimester was prospectively recruited from each cluster. Pregnant women were eligible for inclusion if they had no intention of leaving the study area for the next 2 years and provided informed written consent. Eligible pregnant women were identified through antenatal consultations (women attending their third antenatal visit), and were included until the desired sample size was reached in each health center. After birth, singleton babies without major birth defects were included in the study and followed quarterly until 18 months of age by external nurses not involved in the intervention delivery. Recruitment of study participants occurred from August 6, 2009 through December 29, 2011, and participants were followed until June 27, 2012.

### Intervention

In the control centers, routine preventive, promotional, and curative services were provided to pregnant and lactating women, and children aged <5 years as per national policy.

Monthly growth monitoring and promotion (GMP) of all children below 24 months of age is organized by auxiliary nurses. During these session children are weighed and, if applicable, vaccinated (up to 9 months).

The nutrition counseling intervention was implemented in the intervention centers within the usual care environment. The intervention aimed to: i) improve communication between care providers and women at any contact for prenatal visits and children’s services; and ii) enhance the nutrition component of the existing maternal and child national program, which includes prenatal care, immunization, and healthy and sick child consultations.

Before the study, all healthcare providers in the intervention arm were trained in communication and nutrition at the district hospital in April 2009.

The communication training focused on skills for educating caregivers, and how to listen to and build confidence with caregivers to introduce behavior change. Training was based on the patient-centered model developed by Stewart et al. [37], existing literature [38–41], and the World Health Organization (WHO)/United Nations Children’s Emergency Fund counseling course [42]. This training lasted 1 week and was led by a pediatrician with support from an expert on patient-centered communication from the Health Care Department, PXL University College, Belgium. Training covered three areas of knowledge: activation of medical knowledge, implementation of a framework for family anamnesis based on Gordon’s 11 functional health patterns, and basic communication models [43, 44]. The training used presentation materials, exchange of experiences, and role play. Finally, detailed family history sheets were developed, tested, and implemented by participants. The goal was to produce a tool that allowed extensive assessment of a family’s social structure, living conditions, and domestic habits. These sheets included medical and family background questions for specific pediatric problems related to malnutrition.

Providers were also trained by the study team on maternal and child nutrition. This aimed to improve health providers’ skills in: (i) providing appropriate feeding counseling; ii) assessing child nutritional status and feeding problems; and (iii) making recommendations. This training was, focused on pregnant women’s diet, breastfeeding, complementary feeding practices, and further counseling skills based on the WHO *Manual on Counseling the Mother* and the Integrated Management of Child Illness strategy training manual [42, 45]. Particular attention was directed to communication, including imparting communication skills, detecting feeding problems, and negotiating with the caregiver on possible solutions that could be personalized using locally-adapted feeding recommendations. Two refresher training sessions were organized during the study period.

Individual nutrition counseling was provided to all women attending the intervention centers during pregnancy and the first 18 months of their child’s life. Counseling contacts were scheduled to begin during pregnancy and continue until the child reached 18 months of age. At each contact, health providers used a patient-centered approach to explore the caregiver’s and child’s situation and current feeding practices, and identify their specific needs in terms of nutrition, health advice, and curative care. They then individually counseled caregivers on theirs needs or their child’s age-specific needs, taking into account the household socioeconomic situation and problems identified, allowing the caregiver to choose recommendations that were feasible and acceptable from various options.

During pregnancy, counseling included the reduction of heavy work, advice on the need to rest, use of health services, consumption of iron and folic acid supplementation, and use of malaria prevention. Healthcare providers discussed dietary behavior and explored inadequate practices such as dietary prohibitions and restrictions related to some beliefs with pregnant women individually, using a patient-centered approach. They then oriented their advice to addressing the challenges and problems identified. General counseling themes included increased portion size and meal frequency, splitting meals, and introducing snacks. A second point of attention was dietary diversification, focusing on the consumption of iron-rich foods, protein-rich animal-source foods, or legumes such as beans and lentils. These messages were adapted to caregivers’ financial situations. Counseling on maternal dietary intake was maintained until the last postnatal consultation 6 weeks after delivery.

During the prenatal period, healthcare providers provided counseling on appropriate breastfeeding practices, such as early initiation of breastfeeding, feeding colostrum, exclusive breastfeeding up to the age of 6 months, and timely introduction of complementary foods.

After delivery, a patient-centered approach was used to identify specific individual needs and problems surrounding child feeding. Thereafter, tailored counseling was provided to promote appropriate feeding practices and offer solutions to any nutrition-related problems. Counseling on breastfeeding practices continued during early infancy. Additional focus areas were the timing of the introduction of complementary foods, the nutritional value of foods available at home, and the quality and quantity of complementary foods.

After 6 months of age the nutrition counseling aimed to improve the quality and quantity of complementary foods. Specific counseling included how to blend food products available at home to improve a child’s dietary intake. Health providers asked caregivers to list all ingredients available at home and explained the benefits of each ingredient for the child.

Based on the food available in the child’s household, health providers showed caregivers various recipes based on typical local meals and modified to increase their energy and nutrient density. For example, for porridge, a common complementary food usually made from cereal flour and water, caregivers were taught to add cooking oil or groundnut flour, soybean flour, or dried fish. Other topics included the improvement of food energy-density using household processes such as grain malting, improvement in food taste by fermenting cereals or adding sugar to porridge and so forth. Meat and egg consumption was promoted by showing caregivers the benefits of animal products for the child’s growth.

During child curative consultations, healthcare providers explored the child’s ongoing feeding practices and tailored advice for the treatment of any illness. Finally, caregivers were counseled on recognizing symptoms of common childhood diseases (fever, diarrhea, acute respiratory infection, and so on), how to address these adequately, and how to recognize dangerous signs.

The intervention was integrated into the health centers’ routine activities, and the same actors working for the existing health program were responsible for implementation. No additional topping up was paid to these healthcare providers for intervention delivery that was monitored by the district health team during routine quarterly supervision. The investigators’ role was restricted to the measurement of outcomes for assessment of intervention effects.

### Data collection

Data were collected by trained field workers not involved in the intervention delivery, and who were not blinded to the intervention. Health center appointments were made each month with participating caregivers. At the scheduled follow-up visits (at 3, 6, 9, 12, 15, or 18 months), caregivers were invited to attend the health center for data collection. The day before the data collection visit, a messenger went to the participating women’s homes to remind them of the appointment. Home visits were planned in the study protocol to encourage non-regularly attending mothers to bring their children to the health center, and to measure the actual care and feeding practices of children who had lost weight. However, for budgetary reasons, these visits were not effective.

Caregivers’ behavior and practices were assessed using interviews with structured questionnaires. All questionnaires were pretested before the study started with a sample of mothers not included in the analysis. Questionnaires were administered in the caregiver’s local language. The first questionnaire was administered during the first month following birth and included information on the child and caregiver socioeconomic characteristics, dietary practices during pregnancy, and child delivery characteristics (e.g., whether the child was thin and presence of malformation). We added questions to assess early breastfeeding practices (delay before feeding the child after delivery, whether the child was fed colostrum, and feeding experiences in the first 72 hours).

Women were asked questions about the follow-up of their pregnancy, including the number of completed antenatal visits, and whether they received preventive treatment for malaria and anemia during pregnancy. They were further questioned on their prenatal dietary practices, with emphasis on pregnancy-related changes in diet. The theme of culturally forbidden foods during pregnancy was also addressed. Finally, the adequacy of the intervention was assessed by asking caregivers whether they received counseling related to diet in pregnancy, early breastfeeding, how to breastfeed, and exclusive breastfeeding in the health center during their last contact with a healthcare provider for prenatal care.

At each follow-up visit, caregivers were questioned about their knowledge on infant feeding, as well as the counseling on breastfeeding and complementary feeding they had received. Feeding frequency was assessed by asking caregivers to recall the number of times they fed their children in the 24 hours before the interview in addition to breastfeeding. Semi-quantitative 24-hour dietary recalls were conducted to assess child feeding practices. The caregiver was asked about all the foods the child had consumed since the previous morning and on the morning of the visit. Each consumed meal was deconstructed into foods. Each food was classified in a food group regardless of the amount consumed, unless the food in question was used as a condiment. Seven food groups were included: cereals, roots and tubers; legumes and nuts; dairy products (milk, yogurt, and cheese); meat products (meat, poultry, and offal) and fish; eggs; fruits and vegetables rich in vitamin A; and other fruits and vegetables. The response options were “yes, consumed” (score 1) and “no, not consumed” (score 0).

During follow-up visits, caregivers were asked to recall their child’s morbidity history for the last 2 weeks using an adapted version of the generic Demographic and Health Survey instrument [46]. This was pre-tested in samples of about 20 mothers not belonging to the study in each health center (intervention and control centers).Caregivers who reported that their child had been sick were asked whether the child had suffered from one of the three main symptoms (diarrhea, fever, cough/breathing difficulties) or from other symptoms. Caregivers were questioned about their exposure to counseling on breastfeeding and complementary feeding practices in the health center during their last contact with a healthcare provider to assess the intervention adequacy at each follow-up visit.

In addition, caregivers’ knowledge about danger signs for which she should bring the child to the health center was verified. The seven key signs that caregivers had to spontaneously mention were: the child cannot drink or suck, his/her health deteriorates, she/he develops a fever, the child displays fast breathing, the child has difficulty for breathing, presence of blood in the stools, and difficulty drinking.

Infants’ birth weight was obtained from birth records, measured by health center nurses using baby scales available at the health centers. Health center nurses received training on anthropometric measurements and the baby scales were checked weekly to ensure accuracy.

Children’s anthropometric measurements were taken at each visit following standard procedures [47]. Recumbent length was measured using length boards (Short Productions, Olney, MD, USA) with 0.1 cm precision. Infants were weighed in light clothing using electronic scales with a precision of 100 g (SECA 872, Germany). All measurement instruments were calibrated before each measurement session. All measurements were performed twice. Standardization exercises for anthropometric measurements were conducted during the initial training and were repeated quarterly during the study. At the time of supportive supervision, the degree to which healthcare providers in the intervention clusters applied the training that they received was assessed by the responsible district nutrition physician through observation and interviews with healthcare providers.

### Data management and analysis

Primary outcomes were the cumulative incidence of wasting, and changes in child weight-for-height z-score (WHZ). Secondary outcomes were the women’s prenatal dietary practices, the Infant and Young Child Feeding practices indicators [48] (Table 1), the child’s birth weight, changes in child height-for-age z-score (HAZ), the prevalence of stunting at endpoint, and cumulative incidence of diarrhea, fever, and acute respiratory infection. Data were entered in double by two data clerks using Epidata [49].

**Table 1:** Definitions of indicators related to Infant and Young Child Feeding practices.

Outcomes	Definition
Early initiation of breastfeeding	Proportion of children who were put to the breast within 24 hours of birth
Fed colostrum	Proportion of children who were fed colostrum
Exclusive breastfeeding	Proportion of infants aged 0–5 months who were fed exclusively with breast milk (no water, other liquids, or solids)
Timely introduction of solid, semi-solid, or soft foods	Proportion of children aged 6–8 months who received solid, semi-solid, or soft foods
Minimum meal frequency at 6–8 months	Proportion of breastfed children aged 6–8 months who received at least two meals (apart from breast milk)
Minimum meal frequency at 9–18 months	Proportion of children aged 9–18 months who received at least three meals (apart from breast milk)
Minimum meal frequency at 6–18 months	Proportion of children aged 6–18 months who received the minimum acceptable number of meals, apart from breast milk (combination of the two above)
Minimum dietary diversity	Proportion of children aged 6–18 months who received at least 4 food groups
Fed with improved cereal flour	Proportion of children 6–18m fed with cereal flour with groundnut, fish powder, oil, or soybean added

Household socioeconomic scores were calculated based on assets and main housing characteristics, using the first component of principal component analysis and presented in quintiles [50].

Exposure to prenatal dietary counseling and early breastfeeding, how to breastfeed and exclusive breastfeeding was analyzed as a binary variable (yes/no) using data from the first follow-up visit. GMP attendance during the previous month and the exposure to counselling on complementary feeding practices during GMP sessions were also analyzed as a binary variable (yes/no) using data from the all the follow-up visits. Mother–child pairs were classified as “regular” or “non-regular” depending on whether or not they attended at least four visits (the median number of visits observed over the entire follow-up). A variable summarizing the level of exposure to counseling on complementary feeding was calculated. This variable was the percentage of GMP sessions where the mother received counseling on complementary feeding. This variable was further categorized into three levels: none (0%), low (0–33%) or high (34–100%).

Dietary intake during pregnancy was coded as “0” for “no improvement” if the woman reported no dietary changes in quantity and diversity, and “1” if the woman reported an increase in meal quantity or diversity, or both.

IYCF indicators were calculated from a qualitative 24-hour recall data. A total dietary diversity score was calculated using the recalled list of food items consumed over the previous day. These were summed to create a child dietary diversity score, ranging from 0 to 7. Minimal dietary diversity was defined as the consumption of at least 4 food groups.

Low birth weight was defined as birth weight below 2500 g. WHO 2006 growth standards were used to calculate WHZ and HAZ using the ZSCORE06 Stata command [51]. Children with a WHZ below −2 were considered wasted and those with a HAZ below −2 were considered stunted [47, 52].

Morbidity was recalled over the two weeks before the interview. Diarrhea was defined as three or more liquid or semi-liquid stools in a 24-h period, fever was determined by caregiver report, and acute respiratory infection was defined as a combination of three symptoms (fever, cough, and respiratory distress). Each of the seven danger signs that required a child to visit a health center received a score of “1” if mentioned spontaneously by caregivers and “0” if not. Points were summed to calculate a knowledge score (range 0–7). All analyses were by intention-to-treat. Comparisons between study arms on participants’ baseline characteristics were performed using mixed-effects logistic regression.

For the analyses of one point binary outcomes, we used mixed-effects logistic regression model with cluster pair as a random intercept and intervention nested as a random slope unless stated otherwise. For these analyses, results are reported as odds ratios (OR) and 95% CI. Linear mixed-effects models with cluster pair as a random intercept and intervention nested as a random slope were used to assess the effect of the intervention on continuous outcomes and binary outcomes collected at multiple point. The estimates were expressed as difference of mean or difference of proportions, and were presented with a 95% confidence interval (CI).

All mixed effects models were adjusted for women’s age, parity, education level, and household socioeconomic score. For child related outcomes, we also adjusted for child age and sex.

The effect of the intervention on the children’s continuous anthropometric outcomes (weight, length, WHZ, and HAZ) was analyzed using a linear piecewise mixed-effects model that included cluster pair and child as random effects. Knots were placed at 2, 4, 6, 8, 10, 12, 14, and 16 months of the child’s age. The linear piecewise mixed-effects model was preferred over a linear mixed-effects model after comparing the fit of both models using a likelihood ratio test. The better fit of the latter model was shown in a visual inspection of the graphs, which demonstrated non-linear relationship between child’s age and HAZ, and child’s age and WHZ. We tested the intervention effect on HAZ and WHZ by comparing the linear piecewise mixed effects model with interaction terms between the intervention allocation and the linear age spline terms against a model without interaction terms using a likelihood ratio test [53]. In cases of statistical significance, this would provide evidence of the effect modification over child’s age and therefore the existence of impact of the intervention on the trends of these continuous outcomes. We further tested the intervention effect on the cumulative incidence of wasting, caregiver-reported diarrhea, acute respiratory infection, and fever. We calculated incidence rates for both study arms with CIs derived from a Poisson distribution adjusted for clustering. Incidence rate ratios (IRR) were calculated using generalized linear latent and mixed models (GLLAMM). For this purpose, we fitted Poisson regression models with cluster pair and child as random effects using the Stata GLLAMM. A robust estimation of standard errors was used to relax the assumption of a Poisson distribution for binary data. For the longitudinal outcomes, children contributed person time to the analyses from birth until 18 months. For the morbidity outcomes, observation time was calculated by multiplying the number of effective visits with the recall period of two weeks, which was the reference period for the morbidity history recall at each visit. A child was defined as lost to follow-up at a given visit when he was absent from that visit and all subsequent visits.

To clarify the number of children lost to follow-up and eliminate possible selection bias, an exploratory analysis was performed to compare sociodemographic characteristics between children declared lost to follow-up and those that contributed data until the age of 18 months. Missing data were processed as they were for the main analyses. However, to assess the robustness of our findings, we conducted multiple imputation (n = 50) of missing data using chained equations under the missing at random assumption, for the point outcomes: mother’s exposure to prenatal dietary counseling, counseling on early breastfeeding, counseling on how to breastfeed, counseling on exclusive breastfeeding, diet improvement during pregnancy, early initiation of breastfeeding, fed colostrum, received something else in the first 72 hours, child birth weight, and the endpoint prevalence of stunting.

A *P*-value of 0.05 was considered to indicate statistical significance, and all tests were two-sided. All statistical analyses were conducted using Stata 12.0 (Statacorp, College Station, TX).

### Ethical considerations

Eligible study participants were informed about the study aims and methods. Written informed consent was obtained from all enrolled mothers. The protocol was examined and approved by the Ethics Committee of the Ministry of Health of Burkina Faso (Comité d’éthique pour la Recherche en Santé), deliberation N° 2008–005 on 5 February, 2008.

The Institutional Review Board of Tropical Medicine of Antwerp, Belgium later approved the protocol in Antwerp on 16 April, 2010 (ref: 10074712). Before implementation of the study, we obtained the approval of the local health authorities and the community representative on the health centers’ management committees. The protocol was registered at ClinicalTrials.gov with the identifier NCT01977365. However, an omission by the principal investigator meant that the study was registered after the enrolment of participants had started. All ongoing and related trials for this intervention have been registered

## Results

**Fig 1:**
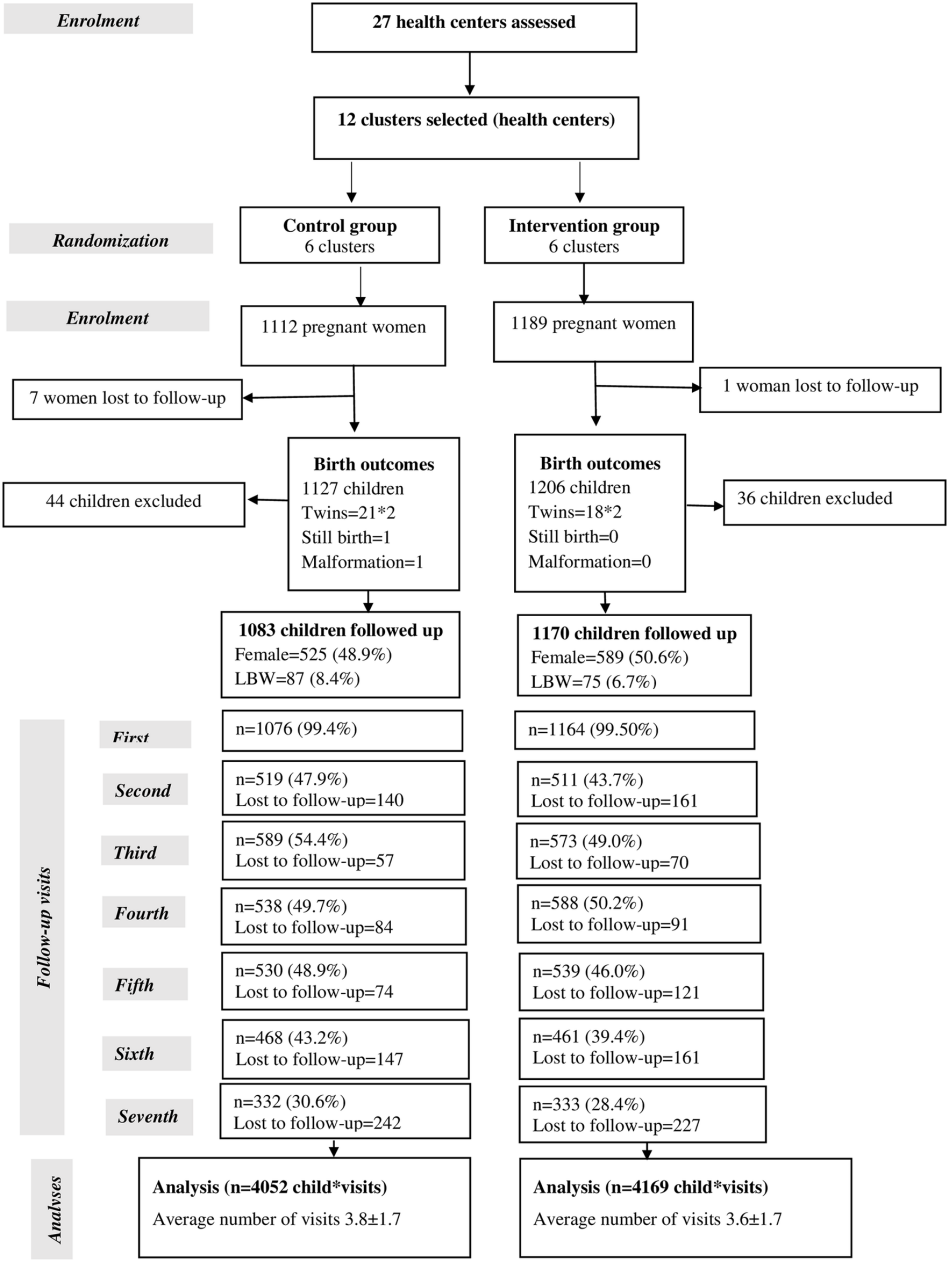
Study profile. LBW, low birth weight.

Between August 2009 and December 2011, 2,301 pregnant women gave informed consent and were enrolled in the study. Eight women were lost to follow-up before delivery. In total, 2,293 pregnant women gave birth to 2,333 children. Eighty children (36 in the intervention arm and 44 in the control arm) were excluded based on non-singleton deliveries (n = 78), malformation that hampered child growth (n = 1), or stillbirth (n = 1). Analyses were conducted with the remaining 2,253 children (1,170 in the intervention and 1,083 in the control arms) (Fig 1). Overall, 1,521 children (67.5%) were classified as lost to follow-up at one point during the follow-up period; 803 (68.6%) in the intervention arm and 718 (66.3%) in the control arm. This pattern varied across visits, with the most lost to follow-up cases recorded at the second, fifth, and sixth visits (Fig 1). No significant difference was found between the two study arms in the distribution of children lost to follow-up.

Comparison of sociodemographic characteristics between children lost to follow-up and those not lost to follow-up showed that the two arms were almost similar, with the exception of the woman’s age (the age groups 14–20 and 31–50 years were more common in the lost to follow-up group), parity (more women with 1–3 parity in the lost to follow-up group), and more women in the lost to follow-up group knew at least one family planning method (S3 Table).

Overall, baseline characteristics were comparable between study arms (Table 2). Although all women attended at least one antenatal consultation, 23.2% did not attend the three antenatal visits recommended by national policy at the study time. Most women started antenatal visits in the first or second trimester. Only 6% of enrolled women did not deliver in a health facility. Nearly 90% of women lived within 5 km of a primary health care center.

**Table 2:** Caregivers’ socioeconomic characteristics and childbirth outcomes by study arm^1^.

Caregiver / household characteristics and childbirth outcomes	Control (N = 1083) n (%)	Intervention (N = 1170) n (%)	*P*-value
**Caregivers’ age group (years)**			
14–20	259 (24.9)	257 (23.1)	
21–30	580 (55.9)	647 (58.3)	0.736^2^
31–50	199 (19.2)	206 (18.6)	
**School attendance**			
None	856 (79.0)	935 (79.9)	
At least primary school	227 (21.0)	235 (20.1)	0.900^3^
**Marital situation**			
Single	10 (0.9)	18 (1.5)	
Monogamous	572 (52.8)	643 (55.0)	0.285^2^
Polygamous	501 (46.3)	508 (43.5)	
**Household socioeconomic score quintile**			
Very poor	246 (22.7)	210 (18.0)	
Poor	218 (20.1)	227 (19.5)	
Intermediate	199 (18.4)	245 (21.0)	0.356^2^
Rich	231 (21.4)	219 (18.8)	
Very rich	188 (17.4)	265 (22.7)	
**Parity (deliveries)**			
1–3	581 (53.7)	627 (53.7)	
4–6	373 (34.4)	412 (35.2)	0.905^2^
≥7	129 (11.9)	130 (11.1)	
**Inter-pregnancy interval <2 years**^4^			
Yes	244 (23.1)	257 (22.9)	0.902^3^
No	811 (76.9)	865 (77.1)	
**Knows at least one family planning method**			
Yes	1036 (95.8)	1111 (95.1)	0.913^3^
No	45 (4.2)	57 (4.9)	
**At least three antenatal visits**			
Yes	823 (76.0)	908 (77.6)	0.447^3^
No	260 (24.0)	262 (22.4)	
**Gestational age at the first antenatal visit**			
First trimester	416 (40.7)	460 (40.9)	
Second trimester	535 (52.4)	574 (51.0)	0.760^2^
Third trimester	70 (6.9)	91 (8.1)	
**Received malaria intermittent preventive treatment in pregnancy**			
Yes	1081 (99.9)	1159 (99.6)	0.162^3^
No	1 (0.1)	5 (0.4)	
**Delivery at a health center**			
Yes	1020 (94.2)	1100 (94.0)	0.694^3^
No	63 (5.8)	70 (6.0)	
**Distance from home to health center**			
≤5 km	909 (90.8)	998 (89.1)	0.845^2^
6–10 km	84 (8.4)	111 (9.9)	
>10 km	8 (0.8)	11 (1.0)	
**Child’s sex**			
Female	525 (48.9)	589 (50.6)	0.429^3^
Male	549 (51.1)	574 (49.4)	

^1^ There are missing data on: i) Maternal age (4.7% missing) this information was collected only from official document such as birth certificate or ID card; ii) Early pregnancy (3.4% missing), in these cases the previous pregnancy were mostly achieved by an abortion; iii) Gestational age at the first antenatal visit (4.8% missing) that was calculated based on the date of the last menstruation as reported by each woman; iv) Distance to the health center (5.9% missing), based on the women declaration and verify from the health center. In case of absence of verification this information was recorded as missing.

^2^ Computed using mixed-effects ordered logistic regression models with health center catchment area as the random effect

^3^ Computed using mixed-effects logistic regression models with health center catchment area as the random effect

^4^ Only multiparous women.

Table 3 shows the comparison of women’s exposure to the different counseling themes between the two arms. Although generally low, exposure to all the counseling themes was significantly higher in the intervention arm. Only 376 (32.2%) pregnant women in the intervention arm effectively received counseling on diet in pregnancy. However, the odds of being exposed to prenatal dietary counseling were almost three times higher in the intervention arm compared with the control arm (OR 2.9; 95% CI: 1.4, 6.0; *P* = 0.004) (Table 3). The difference between the two arms was larger for counseling on complementary feeding during GMP sessions (45.5% vs 18.5%, *P* <0.001). Among follow-up visits for regular women, 24.3% of the intervention arm and 64.2% of the control arm did not receive counseling on complementary feeding at their last contact with health services when their child was aged between 6 and 18 months. However, the intensity of the counseling exposure in GMP sessions was higher in the intervention arm (*P* <0.001) (Table 3).

**Table 3:** Women’s exposure to counseling during pregnancy and childhood^1^.

Outcomes	Included visits	Control		Intervention		OR/ DP/	95% CI	*P*-value
		n	Values	n	Values			
**Exposure to prenatal dietary counseling** % (n) ^2^	Baseline ^5^	1,079	16.1 (174)	1,168	32.2 (376)	2.9	(1.4, 6.0)	0.004^8^
**Exposure to counseling on early breastfeeding** % (n) ^3^	Baseline ^5^	1,080	7.7 (83)	1,167	22.4 (261)	4.8	(2.4, 9.4)	<0.001^8^
**Exposure to counseling on breastfeeding techniques** % (n) ^3^	Baseline ^5^	1,080	4.3 (46)	1,167	11.2 (131)	4.3	(1.5, 11.9)	0.005^8^
**Exposure to counseling on exclusive breastfeeding** % (n) ^3^	Baseline ^5^	1,079	51.1 (551)	1,168	75.7 (884)	3.3	(2.3, 4.9)	<0.001^8^
**Attendance at GMP session the previous month** % (n)	All visits ^6^	4,044	22.9 (928)	4,158	43.0 (1,787)	19.3^7^	(2.5, 36.2)	0.024^9^
**Counseling on complementary feeding practices during the GMP session the previous month** % (n)	All visits ^6^	3,850	18.5 (711)	3,993	45.0 (1,786)	26.3^7^	(17.1, 35.5)	<0.001^9^
**Intensity of counseling on complementary feeding** % (n) ^4^ None Low High	All visits ^6^	510	69.2 (353) 4.3 (22) 26.5 (135)	807	37.6 (303) 6.3 (51) 56.1 (453)	4.5	(2.4, 8.2)	<0.001^8^

^1^ CI, confidence interval; DP, difference of proportion; OR, odds ratio; GMP, growth monitoring and promotion.

^2^ Exposure to prenatal dietary counseling defined as a mother reporting she received dietary counseling at least once during antenatal consultations.

^3^ Exposure to counseling on early breastfeeding, how to breastfeed, and counseling on exclusive breastfeeding were defined as a mother reporting she received the counseling at least once during antenatal consultations and postnatal consultation.

^4^ This variable was the percentage of GMP sessions where the mother received counseling on complementary feeding. This variable was further categorized into three levels of intensity of exposure: none (0%), low (0–33%) or high (34–100%).

^5^ Women were questioned on this variable during the first follow-up visit after delivery.

^6^ Women were questioned on this variable during each follow-up visit after delivery.

^7^ Difference of proportion

^8^ Estimates from a mixed-effects logistic regression model with cluster pair as the random effect and intervention nested as a random slope, adjusted for women’s age, parity, education level, and household socioeconomic score.

^9^ Estimates from a mixed-effects linear regression model with cluster pair as the random effect and intervention nested as a random slope, adjusted for women’s age, parity, education level, and household socioeconomic score.

Among women exposed to counseling on prenatal dietary practices, 9.4% increased their food intake or improved their dietary diversity during pregnancy. The improvement in prenatal dietary practices was significantly more frequent in the intervention arm than the control arm (11.5% *vs*. 7.1%; OR 1.7; 95% CI: 1.2, 2.3; *P *= 0.003) (Table 4).

**Table 4:** Outcomes for dietary practices by intervention arm^1^.

Outcomes on dietary practices	Age range months (Number of visits)	Control		Intervention		OR/DP	95% CI	P-value
		n	Values	n	Values			
**Improved prenatal diet % (n)** ^2^	0–3 (N = 2,253)	1,083	7.1 (77)	1,170	11.5 (134)	1.7	(1.2, 2.3)	0.003^15^
**Early initiation of breastfeeding**^3^	0–3 (N = 2,248)	1,080	98.5 (1,064)	1,168	98.7 (1.153)	1.1	(0.5, 2.4)	0.072^15^
**Fed colostrum** ^4^	0–3 (N = 2,247)	1,080	51.3 (554)	1,167	61.8 (721)	1.6	(1.1, 2.4)	0.032^15^
**Received something else in the first 72 hours** ^5^	0–3 (N = 2,245)	1,079	30.5 (329)	1,166	20.7 (241)	0.6	(0.5, 0.7)	<0.001^15^
**Exclusive breastfeeding** ^6^	0–6 (N = 3,514)	1,713	42.3 (725)	1,801	54.3 (977)	12.8^14^	(2.1, 23.6)	0.020^16^
**Timely introduction of solid, semi-solid, or soft foods** ^7^	6–8 (N = 851)	448	44.6 (200)	403	49.1 (198)	6.8^14^	(-11.3, 24.9)	0.461^16^
**Appropriate age introduction complementary foods** ^8^	0–18 (N = 3,923)	1,921	45.6 (875)	2,002	50.0 (1,000)	3.8^14^	(-0.8, 8.3)	0.105^16^
**Minimum meal frequency at 6–8 months** ^9^	6–8 (N = 878)	459	88.0 (404)	419	93.8 (393)	4.9^14^	(1.1, 8.8)	0.012^16^
**Minimum meal frequency at 9–18 months** ^10^	9–18 (N = 3,454)	1,701	44.1 (750)	1,753	62.8 (1,101)	17.2^14^	(11.0, 23.4)	<0.001^16^
**Minimum meal frequency from 6–18 months** ^11^	6–18 (N = 4,332)	2,160	53.4 (1,154)	2,172	68.8 (1494)	14.1^14^	(9.0, 19.2)	<0.001^16^
**Use of improved cereal flour**^12^	6–18 (N = 4,707)	2,339	29.9 (699)	2,368	55.3 (1310)	24.3^14^	(16.1, 32.6)	<0.001^16^
**Minimum dietary diversity** ^13^	6–18 (N = 4,707)	2,339	22.0 (515)	2,368	28.6 (677)	5.9^14^	(2.7, 9.2)	<0.001^16^

^1^ Data were collected at each follow-up visit by interviews with mothers. The proportions are the answers reported for the number of completed questionnaires for all follow-ups. CI, confidence interval; DP, difference of proportion; OR, odds ratio.

^2^ Improved prenatal diet defined as a mother reporting having increased food intake or more dietary diversity during her pregnancy.

^3^ Defined as the proportion of children who were put to the breast within 24 hours of birth.

^4^ Defined as the proportion of children who were fed colostrum.

^5^ Defined as the proportion of children aged 6–18 months who received something else (including water, semi-solid food, other preparation) in addition to breast milkin the first 72 hours.

^6^ Defined as the proportion of infants aged 0–5 months who were fed exclusively with breast milk (no water, other liquids, or solids).

^7^ Defined as the proportion of children aged 6–8 months who received solid, semi-solid, or soft foods.

^8^ The Woman was asked if the child has already started taking semi-solid food. If so at what age the child started taking semi-solid foods. If the answer is no she was asked at what age she expected to start to give him semi-solid foods. Many cases of missing data on this variable because if the woman answers by “does not know”, the answer was considered as missing.

^9^ Defined as the proportion of breastfed children aged 6–8 months who received at least two meals (apart from breast milk)

^10^ Defined as the proportion of children aged 9–18 months who received at least three meals (apart from breast milk).

^11^ Defined as the proportion of children aged 6–18 months who received the minimum acceptable number of meals (combination of the two above).

^12^ Defined as the proportion of children aged 6–18 months fed with cereal flour with addition of groundnut, fish powder, oil, or soybean flour.

^13^ Defined as the proportion of children aged 6–18 months who received at least 4 food groups.

^14^ Difference of proportion

^15^ Estimates from a mixed-effects logistic regression model with cluster pair as the random effect and intervention nested as a random slope, adjusted for women’s age, parity, education level, and household socioeconomic score.

^16^ Estimates from a mixed-effects linear regression model with cluster pair as the random effect and intervention nested as a random slope, adjusted for women’s age, parity, education level, and household socioeconomic score.

On average, early breastfeeding practices were better in the intervention arm (Table 4). Colostrum was given to the newborn more often in the intervention arm (61.8% *vs* 51.3%; OR 1.6; 95% CI: 1.1, 2.4; *P* = 0.032), while intervention children were less likely to receive other foods in addition to breast milk during the first 72 hours (20.7% *vs* 30.5%; OR 0.6; 95% CI: 0.5, 0.7; *P* <0.001).

Significantly more children in the intervention arm were exclusively breastfed for the first 6 months (54.3% *vs* 42.3; DP 12.8%; 95% CI: 2.1, 23.6; *P* = 0.020). All reported complementary feeding practices were significantly better in the intervention arm, with the exception of timely introduction of solid, semi-solid, or soft foods (Table 4).

**Fig 2:**
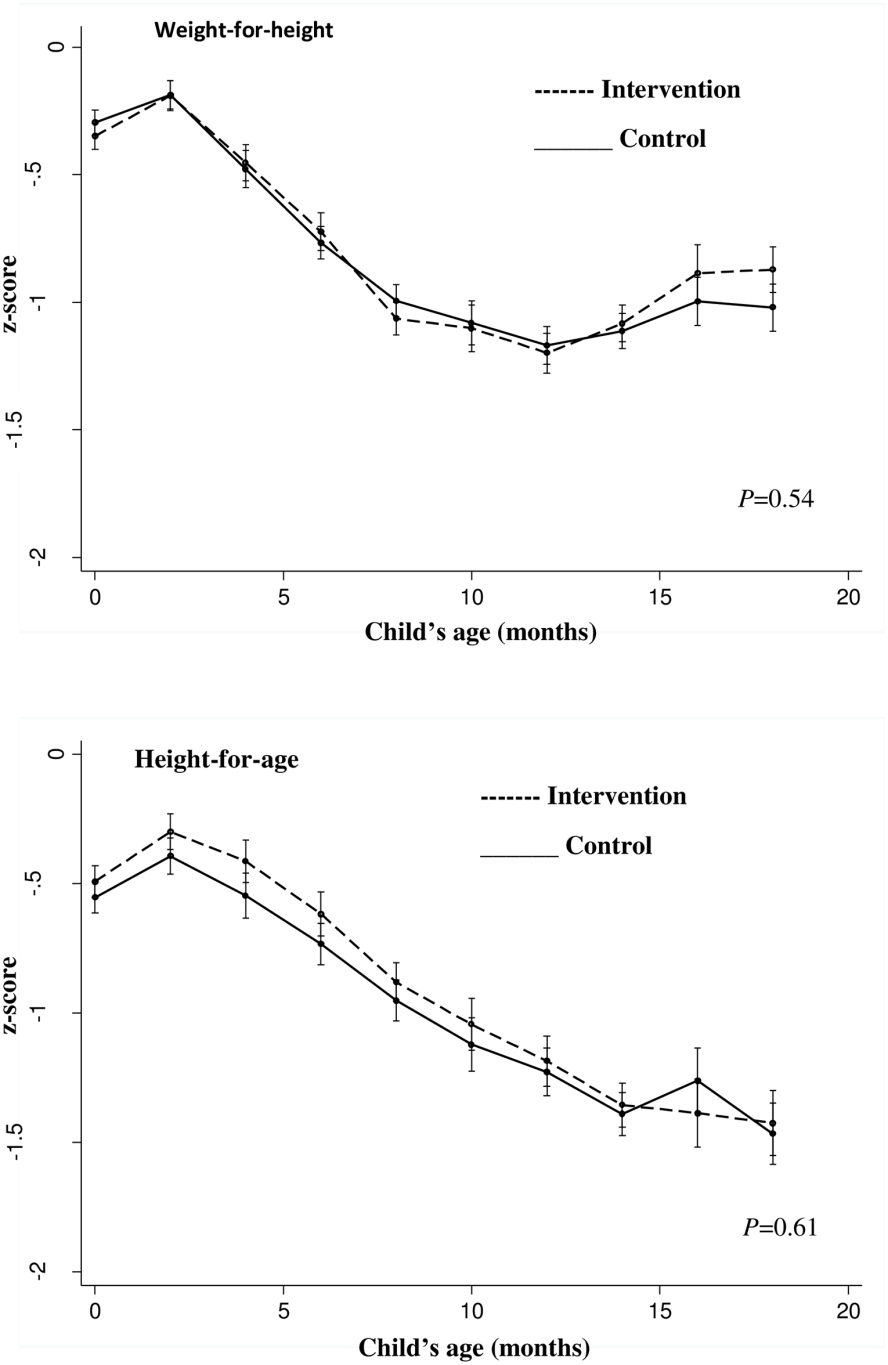
Evolution in modeled mean weight-for-height and height-for-age z-scores at 2-monthly child age intervals by study arm. Estimated using a linear piecewise mixed effects model with cluster pair as the random intercept and intervention nested as a random slope. Models were adjusted for women’s age, parity, education level, child’s sex, and household socioeconomic score. The *P*-values were obtained from a likelihood ratio test comparing a linear piecewise mixed-effects model with main effects for linear spline terms for child’s age against a model with additional interaction terms between intervention allocation and linear spline terms for child’s age. Error bars represent 95% confidence intervals.

The overall median number of completed follow-up visits is 4 visits, with an average of 3.6±1.7 in the intervention arm and 3.8±1.7 in the control arm (*P* = 0.166). Overall, children contributed 32,787 and 33,431 months of follow-up in the intervention and control arms, respectively. Fig 2 shows the effect of the intervention on mean WHZ and HAZ over the entire follow-up period. We found no significant difference in either outcome between the study arms.

The difference in mean birth weight between the study arms was 84.8 g (95% CI: 5.0, 164.5; *P = 0*.*037*), with a higher mean birth weight in the intervention arm compared with the control arm. The intervention arm had a lower low birth weight prevalence (6.8%) compared with the control arm (8.4%), but the difference was not statistically significant (Table 5).

**Table 5:** Effect of the intervention on child growth.

Outcomes	Control	Intervention	*P*-value
**Number of follow up visits (mean** ± SD)	3.8±1.7	3.6±1.7	0.166
**Child birth weight**			
Number of children^1^	1,039	1,111	-
Mean birth weight ± SD	2979±407	3069±444	-
Difference of mean birth weight (95% CI)	Reference	84.8 (5.0, 164.5)	0.037^2^
Proportion of low birth weight % (n)	8.4 (87)	6.8 (75)	-
OR (95% CI)^3^	Reference	0.8 (0.6, 1.1)	0.237
**Wasting**			
Number of observed children × year	2,670	2,719	-
Number of children^1^	1,058	1,144	-
Cumulative episodes of WHZ <−2	490	495	-
Number of episodes of WHZ <−2 per child-year (95% CI)^4^	0.2 (0.2, 0.2)	0.2 (0.2, 0.2)	-
Incidence rate ratio (95% CI)^5^	Reference	1.0 (0.9, 1.2)	0.747
**Stunting**			
Number of children^1^	1,055	1,143	
Prevalence at end point % (n)	13.8 (146)	13.6 (156)	-
OR at end point (95% CI)^6^	Reference	1.0 (0.7, 1.4)	0.898

^1^ Number of children that provided at least one follow-up measurement.

^2^ Estimates from a mixed-effects linear regression model with cluster pair as the random effect and intervention nested as a random slope, adjusted for women’s age, parity, education level, and household socioeconomic score

^3^ Estimates from a mixed-effects logistic regression model with cluster pair as the random effect and intervention nested as a random slope, adjusted for women’s age, parity, education level, and household socioeconomic score.

^4^ CI estimated from a Poisson regression model adjusted for clustering by health center catchment.

^5^ Computed using a generalized linear latent and mixed model, with cluster pair and child as random effects, adjusted for child’s age and sex, women’s age, parity, education level, and household socioeconomic score.

^6^ Computed using a mixed logistic model with cluster pair and child as random effects, adjusted for child’s age and sex, women’s age, parity, education level and household socioeconomic score.

In total, 495 episodes of wasting were recorded in the intervention arm and 490 in the control arm over the entire follow-up period (Table 5). No difference was found in the incidence of wasting between the study arms (IRR 1.0; 95% CI: 0.9, 1.2; *P* = 0.747). We found no significant intervention effect on endpoint stunting prevalence (OR 1.0; 95% CI: 0.7, 1.4; *P* = 0.898) (Table 5).

Overall, the reported mean incidence of child illness in the previous 2 weeks was significantly higher in the intervention arm compared with the control arm (IRR 1.1; 95% CI: 1.0, 1.3, *P* = 0.024). However, no difference was found in the specific incidence of common symptoms (diarrhea, fever, and incidence of acute respiratory infections) between study arms (Table 6). Finally, women in the intervention arm had higher knowledge scores for recognizing dangerous signs relating to child health (*P* = 0.03).

**Table 6:** Effect of the intervention on child morbidity^1^.

Child morbidity outcomes	Control	Intervention	*P*-value
**Reported illness in the 2 weeks before the visit**			
Number of observed children × year^2^	156	160	-
Number of children^3^	1073	1164	
Cumulative episodes of illness (n)	632	730	-
Number of episodes per child × year (95% CI)^4^	4.1 (3.8, 4.4)	4.6 (4.2, 4.9)	-
Incidence rate ratio (95% CI)^5^	reference	1.1 (1.0, 1.3)	0.024
**Reported diarrhea in the 2 weeks before the visit**			
Number of observed children × year^2^	155	160	
Number of children^3^	1068	1160	
Cumulative episodes of diarrhea (n)	202	237	-
Number of episodes per child × year (95% CI)^4^	1.3 (1.1, 1.5)	1.5 (1.3, 1.7)	-
Incidence rate ratio (95% CI)^5^	reference	1.1 (0.9, 1.4)	0.16
**Reported fever in the 2 weeks before the visit**			
Number of observed children × year^2^	155	160	
Number of children^3^	1068	1164	
Cumulative episodes of fever, (n)	542	600	-
Number of episodes per child × year (95% CI)^4^	3.5 (3.2, 3.8)	3.7 (3.5, 4.0)	
Incidence rate ratio (95% CI)^5^	reference	1.1 (0.9, 1.2)	0.15
**Reported acute respiratory infection in the 2 weeks before the visit**			
Number of observed children × year^2^	156	160	
Number of children^3^	1076	1165	
Cumulative episodes of acute respiratory infection (n)	55	52	
Number of episodes per child × year (95% CI)^4^	0.3 (0.3, 0.5)	0.3 (0.2, 0.4)	
Incidence rate ratio (95% CI)^5^	reference	0.9 (0.6, 1.3)	0.61
**Knowledge score for recognizing dangerous child health signs, mean difference, (95% CI)**^6^	reference	3.3 (0.4, 6.4)	0.03

^1^ CI, confidence interval; Child morbidity results based on caregivers’ recall.

^2^ Calculated by the number of children followed-up × number of visits x observation duration of 2 weeks per visit, converted into years.

^3^ Number of children with at least one data point included in the analysis.

^4^ Confidence intervals are estimated from a Poisson regression model adjusted for clustering by health center catchment area.

^5^ Computed using a generalized linear latent and mixed model, with cluster pair and child as random effects, adjusted for child’s age and sex, and household socioeconomic score.

^6^ Computed using a mixed-effects linear regression model, with cluster pair and child as random effects, adjusted for women’s age, parity, education level, and household socioeconomic score.

The sensitivity analysis using multiple imputation of missing data for the point outcomes showed that none of the findings were altered using this larger dataset (S4 Table).

## Discussion

In a rural area of Burkina Faso, training healthcare providers on delivering a personalized educational intervention during usual primary healthcare services to promote appropriate feeding practices for pregnant and lactating women and young children was associated with improvement in pregnant women’s diet, IYCF practices, and increased child birth weight. However, we did not detect significant improvements in child wasting and linear growth during early childhood (up to 18 months of age).

Prenatal nutrition counseling led to a significant improvement in the dietary practices of women in the intervention arm, although appropriateness of dietary intake practices was generally low in both study arms. The study also showed that overall, reported IYCF practices were better in the intervention arm, with the exception of timely introduction of semi-solid or soft food. These results are consistent with preliminary studies that show that it is possible to improve feeding practices through educational interventions by trained health workers embedded in routine preventive services [26–28].

The overall positive results on feeding practices are likely to be due to the specificities of the intervention. Counseling was individual and adapted to each mother–child pair after a thorough analysis of the child’s situation. The fact that counseling was personalized and adapted to the socioeconomic situations of each woman was likely to have contributed to their commitment.

In a health system with limited channels of delivery of community-level health services, it is possible to improve feeding practices, if the few opportunities of contact with caregivers are exploited through an individual communication and counseling approach focused on mother/child-specific needs. However, the overall low proportion of women who effectively improved their dietary practices might be related to the low number of counseling sessions actually received by pregnant women. While over 59% of pregnant women in the intervention arm had started attending antenatal consultations in the second or third trimester, those women could only benefit from a theoretical maximum of two counseling sessions, even if they attended the three prenatal consultations recommended in Burkina Faso health policy at the time of the study. Expanding nutrition counseling to all promotional services targeted to women of childbearing age, such as family planning, or the adjunct of an outreach strategy for early prenatal care may enhance women’s exposure, and lead to improved prenatal dietary practices.

The intervention led to a ~85 g increase in birth weight in the intervention arm compared with the control arm. This result should be interpreted with caution, as birth weight measurements were not fully controlled by study investigators. Nevertheless, healthcare providers working in each health center received training to improve the quality of anthropometric measurements, and the baby scales used for weighing children at birth were checked on a weekly basis. The observed effect on birth weight is consistent with the effect size reported in a review of previous studies on prenatal nutrition counseling [54]. However, as noted in previous studies, we found no significant difference between the two study arms in terms of prevalence of low birth weight [54].

We found no intervention effect on child wasting and linear growth. Although children in the intervention arm had a significantly higher mean birth weight than those in the control arm, the difference in weight narrowed during follow-up despite significant improvement in child feeding practices. To some extent this might be related to the persisting high disease burden during childhood in the study area. According to our 2008 pilot study, 39% of children under 5 years old in the area were sick in the previous 2 weeks. In the present study we did not find any intervention effect on child morbidity. Instead, we observed a higher rate of reported illnesses in the intervention arm, though no specific symptoms were identified as significantly more frequent. This might reflect an issue of over-reporting in this arm, which we discuss below.

Our findings on child growth are inconsistent with some previous studies on the effectiveness of maternal counseling on child anthropometric measurements that reported significant improvements in child linear growth [4, 55, 56] and/or child weight [9, 56, 57]. However, these results should be considered with respect to the content and the context of each intervention.

Most of the previous studies delivered education and behavior change interventions through community-based healthcare providers, whereas our intervention was implemented by facility-based healthcare staff. Lay healthcare providers typically live in the caregiver’s villages, and therefore community-based programs are expected to have a higher coverage compared with the present intervention. The present intervention was modeled on the typical implementation of promotional and preventive services in developing countries. Counseling was delivered to women who spontaneously visited the health centers, and intervention monitoring was performed in health centers. The availability of nutrition services offered in the intervention centers improved, as shown by the higher proportion of women exposed to counseling in these centers compared with the paired control centers. However, we noted that caregivers’ exposure to counseling remained below expectations in the intervention arm. This low exposure to counseling was related to overall low health center attendance. Although six follow-up visits were scheduled for each mother–child pair for the intervention effect measurement, the median number of actual visits was 4 for all the sample, and only 43% of caregivers in the intervention arm attended the scheduled growth monitoring and promotion session the previous month. Suboptimal implementation of the child centered counseling in the intervention centers might also have contributed to the lower exposure to counseling in this arm. Although the intensity of counseling in GMP sessions was significantly higher in the intervention arm, our analysis showed that 24.5% of regular women in the intervention arm who attended GMP sessions did not receive any counseling during that session.

This low coverage reflects the reality of preventive, promotional, and curative services in most developing countries. The advent of free health care for all children under 5 years old and all pregnant women should improve service coverage and may contribute to a better functioning of such interventions. To our knowledge, only one other study conducted in Peru by Penny et al. used health facilities as a delivery platform for a nutritional educational intervention [11]. Unlike our study, that study reported a positive intervention effect on child weight and length. The difference between our results and those of Penny et al. might be explained by differences in basic population characteristics. Considering the major contextual differences between settings, this difference might also be explained by the severity of baseline child malnutrition. First, child wasting was much more prevalent in our setting compared with Peru. Second, food availability linked to a higher dietary diversity was also better in the study area of Penny et al.

The limited availability and accessibility of energy- and nutrient-dense complementary foods in our study area might have downplayed the impact of nutrition counseling on child anthropometry, despite reported improvements in child feeding practices in the intervention arm versus the control arm. Indeed, other dietary intake studies in rural Burkina Faso have reported that children’s and women’s diets are monotonous and poor in micronutrients. The cereals and vegetables that provide the major sources of micronutrients proved inadequate to meet daily nutrient needs for women and children [58]. If the findings of both studies are considered together, it appears that in settings where the availability of good quality complementary foods is lacking, nutrient- and energy-dense supplementary foods should complement nutrition counseling. Another reason for the lack of any intervention effect on child anthropometry in our study could be that the intervention program did not address all determinants of child nutrition and growth. For example, our intervention did not specifically target hygiene and sanitation, two determinants that are strongly implicated in the occurrence of stunting or wasting [59]. According to the latest Demographic and Health Survey, only 25% of children in the study area benefited from hygienic evacuation of the feces in 2010 [46]. Poor hygiene practices are associated with asymptomatic environmental enteric dysfunction, which in turn leads to growth retardation [60, 61].

The strengths of our study include the randomized controlled design and the fact that we measured study outcomes during health center visits to avoid influencing caregivers’ adherence to the intervention through home visits. The intervention occurred during usual health services, and apart from training, the study team did not interfere with service delivery. No additional salary was paid to health workers involved in the intervention implementation. Monitoring was carried out by the health district team. Therefore, the findings can be readily extrapolated to effectiveness settings, and could be validated in other areas with similar health systems. The in-depth approach used in the counseling process was a particular strength of the study.

This study has some weaknesses that warrant attention. We encountered some difficulties in health center matching, given that some community characteristics varied among health centers. The enrollment of a consecutive, convenience sample through antenatal care might have introduced recruitment and selection bias. However, the same recruitment method was used in the intervention and study arms.

The intervention might have reached a limited group of better educated women that had better understanding of the importance of services offered at health centers, which might hamper the external validity of our study.

Data collection on mothers’ exposure to counseling and their feeding practices relied on mothers’ memories, which might have introduced memory bias. However, the same methodology was used for data collection in both study arms. There was also no direct measure of the implementation of the advice given by healthcare workers in individual households. Directly measuring these practices may help to better understand how caregivers’ practices effectively improved following counseling.

Perinatal outcomes regarding stillbirths or congenital abnormalities might have been underestimated in the intervention evaluation. To evaluate the intervention, women were included in the third trimester of pregnancy, meaning that only cases off stillbirth that occurred during this period were counted. Of the 2,301 participating women, eight were lost to follow-up before delivery. We had no information on the characteristics of these births, and those pregnancies could have resulted in stillbirths or congenital abnormalities.

Our study outcomes were analyzed after adjusting for mother’s age, parity, and education level. Unfortunately, we did not have access to data on maternal height, weight, and weight gain during pregnancy, other maternal factors that could also influence birth outcome and child further growth. Finally, we also noted the low participation of children to the health center follow-up visits. As a consequence, there was low data completeness for child outcomes as data was collected during each of the health center visits. However, comparison of sociodemographic characteristics between children declared lost to follow-up and those with more regular health center attendance showed that the two groups were almost comparable. In addition, a multiple imputation analyses of missing data using chained equations under the missing at random assumption, for the point outcomes supported the robustness of our findings.

## Conclusion

Training primary healthcare providers to provide a facility-based patient-centered educational intervention to promote good feeding practices for pregnant and lactating women and young children was associated with improved IYCF practices, and increased child birth weight. However, there was no long-term impact on children’s growth and nutrition status. More comprehensive intervention packages that address feeding practices combined with partial supplementation with energy- and micronutrient-dense complementary foods, behavior communication for change on hygiene and sanitation, and improvement in the overall coverage and quality of primary healthcare are paths to be explored.

## Supporting information

S1 Table Characteristics of health centers used for matching. ^1^ Socioeconomic score based on housing characteristics and assets owned in the household, using principal component analysis. The percentages are proportions of households in the top quintile.
(DOCX)

S2 Table Characteristics of the 27 first-level functional health centers in the Houndé health district in 2008 that guided selection of the 12 participating health centers. ^1^ Health centers were graded using a Microsoft Excel worksheet based on: human resource availability, performance on healthy infant consultations, and performance on measles immunization coverage. After this classification, each health center was visited by the research team to present the project and determine willingness to participate in this formative research. The sample of 12 centers was selected after this exercise.
^2^ Human resources score was calculated by summing the number of professional categories serving at the health center at the time of the survey.
^3^ First performance indicator for health centers. During the survey, we collected data on the total number of children aged under 24 months who had received a healthy child consultation the previous month. The number varied from 0 to 129, with an average of 38±37. This variable was categorized in tertiles, with 0 as the lowest tertile, 1 as the second tertile, and 2 as the third tertile.
^4^ Second performance indicator for health centers. This variable represented the rate of measles vaccine coverage. The rate varied from 50% to 122%, with an average of 91.8%±13.0%. This variable was categorized in tertiles, with 0 as the lowest tertile, 1 as the second tertile, and 2 as the third tertile.
(DOCX)

S3 Table Caregivers’ socioeconomic characteristics and childbirth outcomes between lost to follow-up and children that contributed data until the age of 18 months. ^1^ Computed using mixed-effects ordered logistic regression models with health center catchment area as the random effect
^2^ Computed using mixed-effects logistic regression models with health center catchment area as the random effect
^3^ Only multiparous women.
(DOCX)

S4 Table Women’s exposure to counseling, effect of the intervention on dietary practices, child birth weight and endpoint prevalence of stunting^1^. ^1^ For each outcome, the first line report results for analyses without imputation, the second line report the results after multiple imputation (n = 50) of missing data using chained equations under the missing at random assumption.
(DOCX)

S1 File The study proposal. (DOC)

S2 File CONSORT 2010 checklist. (PDF)

## References

[1] Lartey A. Maternal and child nutrition in Sub-Saharan Africa: challenges and interventions. Proc Nutr Soc. 2008;67(1):105–8. Epub 2008/02/01. 10.1017/S0029665108006083 S0029665108006083. . 18234138

[2] Ahmed T, Hossain M, Sanin KI. Global burden of maternal and child undernutrition and micronutrient deficiencies. Ann Nutr Metab. 2012;61 Suppl 1:8–17. Epub 2013/02/21. 10.1159/000345165 000345165. . 23343943

[3] Dewey KG. The challenges of promoting optimal infant growth. J Nutr. 2001;131(7):1879–80. Epub 2001/07/04. . 11435501 10.1093/jn/131.7.1879

[4] Bhandari N, Mazumder S, Bahl R, Martines J, Black RE, Bhan MK. An educational intervention to promote appropriate complementary feeding practices and physical growth in infants and young children in rural Haryana, India. J Nutr. 2004;134(9):2342–8. Epub 2004/08/31. . 15333726 10.1093/jn/134.9.2342

[5] Patel A, Pusdekar Y, Badhoniya N, Borkar J, Agho KE, Dibley MJ. Determinants of inappropriate complementary feeding practices in young children in India: secondary analysis of National Family Health Survey 2005–2006. Matern Child Nutr. 2012;8 Suppl 1:28–44. Epub 2011/12/30. 10.1111/j.1740-8709.2011.00385.x . 22168517 PMC6860525

[6] Moore AC, Akhter S, Aboud FE. Responsive complementary feeding in rural Bangladesh. Soc Sci Med. 2006;62(8):1917–30. Epub 2005/10/15. 10.1016/j.socscimed.2005.08.058 . 16223552

[7] Ota E, Tobe-Gai R, Mori R, Farrar D. Antenatal dietary advice and supplementation to increase energy and protein intake. Cochrane Database Syst Rev. 2012;9:CD000032 Epub 2012/09/14. 10.1002/14651858.CD000032.pub2 . 22972038

[8] Ota E, Hori H, Mori R, Tobe-Gai R, Farrar D. Antenatal dietary education and supplementation to increase energy and protein intake. Cochrane Database Syst Rev. 2015;6:CD000032 Epub 2015/06/03. 10.1002/14651858.CD000032.pub3 . 26031211 PMC12634316

[9] Bhutta ZA, Ahmed T, Black RE, Cousens S, Dewey K, Giugliani E, et al What works? Interventions for maternal and child undernutrition and survival. Lancet. 2008;371(9610):417–40. Epub 2008/01/22. 10.1016/S0140-6736(07)61693-6 S0140-6736(07)61693-6. . 18206226

[10] Pelto GH, Santos I, Goncalves H, Victora C, Martines J, Habicht JP. Nutrition counseling training changes physician behavior and improves caregiver knowledge acquisition. J Nutr. 2004;134(2):357–62. Epub 2004/01/30. . 14747672 10.1093/jn/134.2.357

[11] Penny ME, Creed-Kanashiro HM, Robert RC, Narro MR, Caulfield LE, Black RE. Effectiveness of an educational intervention delivered through the health services to improve nutrition in young children: a cluster-randomised controlled trial. Lancet. 2005;365(9474):1863–72. Epub 2005/06/01. S0140-6736(05)66426-4 [pii] 10.1016/S0140-6736(05)66426-4 . 15924983

[12] Glenton C, Colvin CJ, Carlsen B, Swartz A, Lewin S, Noyes J, et al Barriers and facilitators to the implementation of lay health worker programmes to improve access to maternal and child health: qualitative evidence synthesis. Cochrane Database Syst Rev. 2013;10:CD010414 Epub 2013/10/09. 10.1002/14651858.CD010414.pub2 . 24101553 PMC6396344

[13] Haines A, Sanders D, Lehmann U, Rowe AK, Lawn JE, Jan S, et al Achieving child survival goals: potential contribution of community health workers. Lancet. 2007;369(9579):2121–31. Epub 2007/06/26. S0140-6736(07)60325-0 [pii] 10.1016/S0140-6736(07)60325-0 . 17586307

[14] Lewin S, Munabi-Babigumira S, Glenton C, Daniels K, Bosch-Capblanch X, van Wyk BE, et al Lay health workers in primary and community health care for maternal and child health and the management of infectious diseases. Cochrane Database Syst Rev. 2010;(3):CD004015 Epub 2010/03/20. 10.1002/14651858.CD004015.pub3 . 20238326 PMC6485809

[15] Gilmore B, McAuliffe E. Effectiveness of community health workers delivering preventive interventions for maternal and child health in low- and middle-income countries: a systematic review. BMC Public Health. 2013;13:847 Epub 2013/09/17. 10.1186/1471-2458-13-847 ; 24034792 PMC3848754

[16] Kok MC, Kane SS, Tulloch O, Ormel H, Theobald S, Dieleman M, et al How does context influence performance of community health workers in low- and middle-income countries? Evidence from the literature. Health Res Policy Syst. 2015;13:13 Epub 2015/04/19. 10.1186/s12961-015-0001-3 ; 25890229 PMC4358881

[17] Bryce J, Coitinho D, Darnton-Hill I, Pelletier D, Pinstrup-Andersen P. Maternal and child undernutrition: effective action at national level. Lancet. 2008;371(9611):510–26. Epub 2008/01/22. 10.1016/S0140-6736(07)61694-8 S0140-6736(07)61694-8. . 18206224

[18] Lutter CK, Daelmans BM, de Onis M, Kothari MT, Ruel MT, Arimond M, et al Undernutrition, poor feeding practices, and low coverage of key nutrition interventions. Pediatrics. 2011;128(6):e1418–27. Epub 2011/11/09. 10.1542/peds.2011-1392 . 22065267

[19] Haroon S, Das JK, Salam RA, Imdad A, Bhutta ZA. Breastfeeding promotion interventions and breastfeeding practices: a systematic review. BMC Public Health. 2013;13 Suppl 3:S20 Epub 2014/02/26. 10.1186/1471-2458-13-S3-S20 1471-2458-13-S3-S20 ; 24564836 PMC3847366

[20] Black RE, Victora CG, Walker SP, Bhutta ZA, Christian P, de Onis M, et al Maternal and child undernutrition and overweight in low-income and middle-income countries. Lancet. 2013;382(9890):427–51. Epub 2013/06/12. 10.1016/S0140-6736(13)60937-X S0140-6736(13)60937-X . 23746772

[21] Prendergast AJ, Humphrey JH. The stunting syndrome in developing countries. Paediatr Int Child Health. 2014;34(4):250–65. Epub 2014/10/14. 10.1179/2046905514Y.0000000158 ; 25310000 PMC4232245

[22] de Onis M, Dewey KG, Borghi E, Onyango AW, Blossner M, Daelmans B, et al The World Health Organization's global target for reducing childhood stunting by 2025: rationale and proposed actions. Matern Child Nutr. 2013;9 Suppl 2:6–26. Epub 2013/10/23. 10.1111/mcn.12075 . 24074315 PMC6860845

[23] WHO. Building a future for women and children. The 2012 report. Geneva, Switzerland: World Health Organization; 2012.

[24] Roberfroid D, Pelto GH, Kolsteren P. Plot and see! Maternal comprehension of growth charts worldwide. Trop Med Int Health. 2007;12(9):1074–86. Epub 2007/09/19. TMI1890 [pii] 10.1111/j.1365-3156.2007.01890.x . 17875018

[25] Roberfroid D, Lefevre P, Hoeree T, Kolsteren P. Perceptions of growth monitoring and promotion among an international panel of district medical officers. J Health Popul Nutr. 2005;23(3):207–14. Epub 2005/11/03. . 16262016

[26] Imdad A, Yakoob MY, Bhutta ZA. Impact of maternal education about complementary feeding and provision of complementary foods on child growth in developing countries. BMC Public Health. 2011;11 Suppl 3:S25 Epub 2011/04/29. 10.1186/1471-2458-11-S3-S25 1471-2458-11-S3-S25 ; 21501443 PMC3231899

[27] Sunguya BF, Poudel KC, Mlunde LB, Shakya P, Urassa DP, Jimba M, et al Effectiveness of nutrition training of health workers toward improving caregivers' feeding practices for children aged six months to two years: a systematic review. Nutr J. 2013;12:66 Epub 2013/05/22. 10.1186/1475-2891-12-66 1475-2891-12-66 ; 23688174 PMC3668136

[28] Sunguya BF, Poudel KC, Mlunde LB, Urassa DP, Yasuoka J, Jimba M. Nutrition training improves health workers' nutrition knowledge and competence to manage child undernutrition: a systematic review. Front Public Health. 2013;1:37 Epub 2013/12/19. 10.3389/fpubh.2013.00037 ; 24350206 PMC3859930

[29] Institute on Medicine. "Crossing the Quality Chasm: A New Health System for the 21st Century". Retrieved 26 November 2012.

[30] Cegala DJ, Marinelli T, Post D. The effects of patient communication skills training on compliance. Arch Fam Med. 2000;9(1):57–64. Epub 2000/02/09. . 10664643 10.1001/archfami.9.1.57

[31] Little P, Everitt H, Williamson I, Warner G, Moore M, Gould C, et al Preferences of patients for patient centred approach to consultation in primary care: observational study. BMJ. 2001;322(7284):468–72. Epub 2001/02/27. . 11222423 10.1136/bmj.322.7284.468 PMC26564

[32] Huybregts L, Roberfroid D, Lanou H, Menten J, Meda N, Van Camp J, et al Prenatal food supplementation fortified with multiple micronutrients increases birth length: a randomized controlled trial in rural Burkina Faso. Am J Clin Nutr. 2009;90(6):1593–600. Epub 2009/10/09. 10.3945/ajcn.2009.28253 ajcn.2009.28253. . 19812173

[33] Roberfroid D, Huybregts L, Lanou H, Henry MC, Meda N, Menten J, et al Effects of maternal multiple micronutrient supplementation on fetal growth: a double-blind randomized controlled trial in rural Burkina Faso. Am J Clin Nutr. 2008;88(5):1330–40. Epub 2008/11/11. 88/5/1330 [pii]. . 18996870 10.3945/ajcn.2008.26296

[34] Ministère de la sante, Secrétariat Général, Direction générale des études et des statistiques sectorielles. Annuaires statistiques 2014. Burkina Faso. 330p. http://cns.bf/img/pdf/annuaire_2014_du_ms.pdf. 2014.

[35] Programme Alimentaire Mondial des Nations Unies (PAM), Service de l’Analyse de la Sécurité Alimentaire (VAM) Analyse Globale de la Vulnérabilité, de la Sécurité Alimentaire et de la Nutrition (AGVSAN). Siège social: Via CG Viola 68, Parco de Medici, 00148, Rome, Italie http://documentswfporg/stellent/groups/public/documents/ena/wfp266835pdf

[36] Hayes RJ, Bennett S. Simple sample size calculation for cluster-randomized trials. Int J Epidemiol. 1999;28(2):319–26. Epub 1999/05/26. 10342698 10.1093/ije/28.2.319

[37] Stewart M. Patient-centered medicine: transforming the clinical method.. Radcliffe Publishing, 2003.

[38] D'Ivernois JF, Gagnayre R. Apprendre à éduquer le patient Approche pédagogique (2 e édition), Maloine, Paris (2004).

[39] Gordon M, Collet C. Diagnostic infirmier: méthodes et applications. Medsi-McGraw-Hill, 1991. 1991.

[40] andolo CGpdlcalpt, art et erreurs de la communication. Elsevier Masson, 2007. Guide pratique de la communication avec le patient: techniques, art et erreurs de la communication. Elsevier Masson, 2007. 2007.

[41] Rouquette C. Education et conseils aux patients Les fondamentux. Editions Lamarre 2004.

[42] WHO, UNICEF. *Infant and Young Child Feeding Counseling*: *An Integrated Course*. World Health Organization 2006.

[43] Gordon M. Nursing diagnosis: Process and application. 3rd ed ed. St. Louis: Mosby 1994.

[44] Iandolo C. Guide pratique de la communication avec le patient.2001.

[45] WHO UNICEF. HANDBOOK: Integrated Management of Childhood Illness Department of Child and Adolescent Health and Development (CAH). World Health Organization; 2005.

[46] Institut National de la Statistique et de la Démographie (INSD) Ministère de l'Économie et des Finances O, Burkina Faso et ICF International, Calverton, Maryland, USA. Enquête Démographique et de Santé (EDS-BF IV). Burkina FasoAvril 2012.

[47] Gibson RS. Principles of nutritional assessment. New York, NY: Oxford University Press, 1990.

[48] World Health Organization. Indicators for assessing infant and young child feeding practices. Part I: definition. ISBN: 9789241596664. 2008:26 pages.

[49] Lauritsen J BM. EpiData (version 3) The EpiData Association. 2003–2004;Odense, Denmark.

[50] Vyas S, Kumaranayake L. Constructing socio-economic status indices: how to use principal components analysis. Health policy and planning. 2006;21(6):459–68. 10.1093/heapol/czl029 . 17030551

[51] Leroy JL. (2011). ZSCORE06: Stata command for the calculation of anthropometric z-scores using the 2006 WHO child growth standards [computer program]. http://econpapers.repec.org/software/bocbocode/s457279.htm. Accessed 11 September 2015.

[52] World Health Organization. WHO child growth standards: methods and development. Geneva, Switzerland: WHO, 2006.

[53] Fitzmaurice G. M., Laird N. M., & Ware J. H. Applied Longitudinal Analysis. Hoboken, NJ: Wiley; 2004.

[54] Girard AW, Olude O. Nutrition education and counselling provided during pregnancy: effects on maternal, neonatal and child health outcomes. Paediatr Perinat Epidemiol. 2012;26 Suppl 1:191–204. Epub 2012/07/07. 10.1111/j.1365-3016.2012.01278.x . 22742611

[55] Bhandari N, Bahl R, Mazumdar S, Martines J, Black RE, Bhan MK. Effect of community-based promotion of exclusive breastfeeding on diarrhoeal illness and growth: a cluster randomised controlled trial. Lancet. 2003;361(9367):1418–23. Epub 2003/05/03. S0140-6736(03)13134-0 [pii] 10.1016/S0140-6736(03)13134-0 . 12727395

[56] Shi L, Zhang J, Wang Y, Caulfield LE, Guyer B. Effectiveness of an educational intervention on complementary feeding practices and growth in rural China: a cluster randomised controlled trial. Public Health Nutr. 2010;13(4):556–65. Epub 2009/08/27. 10.1017/S1368980009991364 S1368980009991364. . 19706219

[57] Guldan GS, Fan HC, Ma X, Ni ZZ, Xiang X, Tang MZ. Culturally appropriate nutrition education improves infant feeding and growth in rural Sichuan, China. J Nutr. 2000;130(5):1204–11. Epub 2000/05/10. . 10801920 10.1093/jn/130.5.1204

[58] Arsenault JE, Nikiema L, Allemand P, Ayassou KA, Lanou H, Moursi M, et al Seasonal differences in food and nutrient intakes among young children and their mothers in rural Burkina Faso. J Nutr Sci. 2014;3:e55 Epub 2014/01/01. 10.1017/jns.2014.53 ; 26101623 PMC4473133

[59] Rah JH, Cronin AA, Badgaiyan B, Aguayo VM, Coates S, Ahmed S. Household sanitation and personal hygiene practices are associated with child stunting in rural India: a cross-sectional analysis of surveys. BMJ Open. 2015;5(2):e005180 Epub 2015/02/14. 10.1136/bmjopen-2014-005180 bmjopen-2014-005180. ; 25678539 PMC4330332

[60] Mbuya MN, Humphrey JH. Preventing environmental enteric dysfunction through improved water, sanitation and hygiene: an opportunity for stunting reduction in developing countries. Matern Child Nutr. 2015 Epub 2015/11/07. 10.1111/mcn.12220 . 26542185 PMC5019251

[61] Humphrey JH. Child undernutrition, tropical enteropathy, toilets, and handwashing. Lancet. 2009;374(9694):1032–5. Epub 2009/09/22. 10.1016/S0140-6736(09)60950-8 . 19766883

