# Supplementary material for: Effectiveness of facility-based personalized maternal nutrition counseling in improving child growth and morbidity up to 18 months: A cluster-randomized controlled trial in rural Burkina Faso
Source: PLoS One. 2017 May 25;12(5):e0177839. doi: 10.1371/journal.pone.0177839 (PMC5444625; doi:10.1371/journal.pone.0177839)
Supplement: S1 Table — 1 Socioeconomic score based on housing characteristics and assets owned in the household, using principal component analysis. The percentages are proportions of households in the top quintile. (DOCX) [file pone.0177839.s001.docx]

S1 Table. Characteristics of the 27 first-level functional health centers in the Houndé health district in 2008 that guided selection of the 12 participating health centers^1^.

| **Health centers** | **Human^2^ resources** | **Performance of Growth Monitoring and Promotion^3^** | **Measles immunization rate^4^** |
| --- | --- | --- | --- |
| URBAIN DE HOUNDE | 13 | 2 | 2 |
| INDINI | 9 | 2 | 1 |
| BONI | 8 | 1 | 1 |
| KOUMBIA | 7 | - | 1 |
| WAKUY | 7 | 1 | 2 |
| FOUNZAN | 7 | 0 | 1 |
| PE | 6 | - | 2 |
| DOUGOUMATO II | 6 | - | 2 |
| BOUERE | 6 | - | 1 |
| BEKUY | 6 | 2 | 0 |
| KOTI | 6 | 2 | 0 |
| BOUAHOUN | 6 | 1 | 2 |
| KARI | 6 | 1 | 2 |
| BEREBA | 6 | 1 | 1 |
| KARABA | 6 | 1 | 1 |
| FAFO | 6 | 1 | 0 |
| SARA | 6 | 1 | 0 |
| BOHOKARI | 5 | 2 | 2 |
| NAHI | 5 | 0 | 2 |
| BASSE | 4 | - | 2 |
| KOULAHO | 4 | - | 0 |
| KIERE | 4 | 2 | 0 |
| KOVIO | 4 | 0 | - |
| DOHOUN | 4 | 0 | 2 |
| GOMBELEDOUGOU | 4 | 0 | 1 |
| MARO | 4 | 0 | 0 |
| SEBEDOUGOU | 4 | 0 | 0 |

^1^ Health centers were graded using a Microsoft Excel worksheet based on: human resource availability, performance on healthy infant consultations, and performance on measles immunization coverage. After this classification, each health center was visited by the research team to present the project and determine willingness to participate in this formative research. The sample of 12 centers was selected after this exercise.

^2^ Human resources score was calculated by summing the number of professional categories serving at the health center at the time of the survey.

^3^ First performance indicator for health centers. During the survey, we collected data on the total number of children aged under 24 months who had received a healthy child consultation the previous month. The number varied from 0 to 129, with an average of 38±37. This variable was categorized in tertiles, with 0 as the lowest tertile, 1 as the second tertile, and 2 as the third tertile.

^4^ Second performance indicator for health centers. This variable represented the rate of measles vaccine coverage. The rate varied from 50% to 122%, with an average of 91.8%±13.0%. This variable was categorized in tertiles, with 0 as the lowest tertile, 1 as the second tertile, and 2 as the third tertile.
