# Supplementary material for: Effectiveness of facility-based personalized maternal nutrition counseling in improving child growth and morbidity up to 18 months: A cluster-randomized controlled trial in rural Burkina Faso
Source: PLoS One. 2017 May 25;12(5):e0177839. doi: 10.1371/journal.pone.0177839 (PMC5444625; doi:10.1371/journal.pone.0177839)
Supplement: S3 Table — 1 Computed using mixed-effects ordered logistic regression models with health center catchment area as the random effect 2 Computed using mixed-effects logistic regression models with health center catchment area as the random effect 3 Only multiparous women. (DOCX) [file pone.0177839.s003.docx]

S3 Table. Caregivers’ socioeconomic characteristics and childbirth outcomes between lost to follow-up and children that contributed data until the age of 18 months^1^.

|  | **Lost to follow-up** | | |
| --- | --- | --- | --- |
| **Caregiver / household characteristics and childbirth outcomes** | **NO (n=732)**  **n (%)** | **YES (n=1521)**  **n (%)** | **P-value** |
| **Caregivers’ age group (years)**  14–20  21–30  31–50 | 142 (20.9)  415 (61.0)  123 (18.1) | 374 (25.5)  812 (55.3)  282 (19.2) | 0.029 |
| **School attendance**  None  At least primary school | 585 (79.9)  147 (20.1) | 1206 (79.3)  315 (20.7) | 0.729 |
| **Marital situation**  Single  Monogamous  Polygamous | 06 (0.8)  390 (53.3)  336 (45.9) | 22 (1.4)  825 (54.3)  673 (44.3) | 0.378 |
| **Household socioeconomic score quintile**  Very poor  Poor  Intermediate  Rich  Very rich | 151 (20.6)  143 (19.5)  150 (20.5)  139 (18.9)  149 (20.4) | 305 (20.1)  302 (19.9)  294 (19.4)  311 (20.5)  304 (20.1) | 0.910 |
| **Parity (deliveries)**  1–3  4–6  ≥7 | 366 (50.0)  279 (38.1)  87 (11.9) | 842 (55.4)  506 (33.3)  172 (11.3) | 0.047 |
| **Inter-pregnancy interval <2 years**^2^  Yes  No | 157 (22.0)  556 (78.0) | 344 (23.5)  1120 (76.5) | 0.442 |
| **Knows at least one family planning method**  Yes  No | 686 (93.8)  45 (6.2) | 1461 (96.2)  57 (3.8) | 0.010 |
| **At least three antenatal visits**  Yes  No | 559 (76.4)  173 (23.6) | 1172 (77.0)  349 (23.0) | 0.717 |
| **Gestational age at the first antenatal visit**  First trimester  Second trimester  Third trimester | 278 (39.4)  373 (52.9)  54 (7.7) | 598 (41.5)  736 (51.1)  107 (7.4) | 0.658 |
| **Received malaria intermittent preventive treatment in**  **pregnancy**  Yes  No | 727 (99.7)  2 (0.3) | 1513 (99.7)  4 (0.3) | 0.963 |
| **Delivery at a health center**  Yes  No | 693 (94.7)  39 (5.3) | 1427 (93.8)  94 (6.2) | 0.421 |
| **Distance from home to health center**  ≤5 km  6–10 km  >10 km | 620 (89.9)  68 (9.8)  2 (0.3) | 1287 (89.9)  127 (8.9)  17 (1.2) | 0.096 |
| **Child’s sex**  Female  Male | 366 (50.3)  361 (49.7) | 748 (49.5)  762 (50.5) | 0.721 |
| **Low birth weight**  Yes  No | 48 (6.8)  653 (93.2) | 114 (7.9)  1335 (92.1) | 0.400 |

^1^ A child was defined as lost to follow-up at a given visit when he was absent from that visit and all subsequent visits.

^2^ Only multiparous women.
