# Supplementary material for: Effectiveness of facility-based personalized maternal nutrition counseling in improving child growth and morbidity up to 18 months: A cluster-randomized controlled trial in rural Burkina Faso
Source: PLoS One. 2017 May 25;12(5):e0177839. doi: 10.1371/journal.pone.0177839 (PMC5444625; doi:10.1371/journal.pone.0177839)
Supplement: S4 Table — 1 For each outcome, the first line report results for analyses without imputation, the second line report the results after multiple imputation (n = 50) of missing data using chained equations under the missing at random assumption. (DOCX) [file pone.0177839.s004.docx]

S4 Table. Women’s exposure to counseling during pregnancy, effect of the intervention on women and child’s dietary practices, child birth weight and endpoint prevalence of stunting^1^

|  | **Coefficient** | **Standard error** | **Estimate** | **95% CI** | **P-value** |
| --- | --- | --- | --- | --- | --- |
| **Exposure to prenatal dietary counseling** | 1.06 | 0.37 | 2.85 | (0.33, 1.80) | 0.004^2^ |
|  | 1.07 | 0.37 | 2.88 | (0.34, 1.80) | 0.004^2^ |
| **Exposure to counseling on early breastfeeding** | 1.56 | 0.34 | 4.57 | (0.89, 2.23) | <0.001^2^ |
|  | 1.55 | 0.32 | 4.74 | (0.91, 2.19) | <0.001^2^ |
| **Exposure to counseling on how to breastfeed** | 1.45 | 0.52 | 2.80 | (0.43, 2.47) | 0.005^2^ |
|  | 1.32 | 0.43 | 3.02 | (0.47, 2.19) | 0.002^2^ |
| **Exposure to counseling on exclusive breastfeeding % (n)** | 1.20 | 0.19 | 6.28 | (0.83, 1.58) | <0.001^2^ |
|  | 1.17 | 0.19 | 6.10 | (0.78, 1.56) | <0.001^2^ |
| **Improved prenatal diet** | 0.51 | 0.17 | 3.03 | 0.18, 0.85) | 0.002^2^ |
|  | 0.50 | 0.17 | 3.00 | (0.18, 0.84) | 0.003^2^ |
| **Early initiation of breastfeeding3** | 0.13 | 0.37 | 0.36 | (-0.60, 0.87) | 0.720^2^ |
|  | 0.14 | 0.36 | 0.39 | (-0.57, 0.85) | 0.697^2^ |
| **Fed colostrum** | 0.45 | 0.21 | 2.15 | (0.40, 0.87) | 0.032^2^ |
|  | 0.48 | 0.21 | 2.29 | (0.07, 0.88) | 0.022^2^ |
| **Received something else in the first 72 hours** | -0.58 | 0.11 | -5.24 | (-0.80, -0.36) | <0.001^2^ |
|  | -0.51 | 0.15 | -3.45 | (-0.80, -0.22) | 0.001^2^ |
| **Child birth weight, g (mean ± SD)** | 84.69 | 40.72 | 2.08 | (4.89,164.50) | 0.040^3^ |
|  | 82.00 | 37.96 | 2.16 | (7.59, 156.41) | 0.031^3^ |
| **Low birth weight % (n)** | -0.20 | 0.17 | -1.20 | (-0.54, 0.13) | 0.229^2^ |
|  | -0.09 | 0.23 | -0.40 | (-0.55, -0.36) | 0.690^2^ |
| **Stunting** | -0.04 | 0.18 | -0.23 | (-0.40, 0.31) | 0.814^2^ |
|  | 0.02 | 0.12 | 0.21 | (-0.21, 0.26) | 0.833^2^ |

^1^ For each outcome, the first line report results for analyses without imputation, the second line report the results after multiple imputation (n=50) of missing data using chained equations under the missing at random assumption.

^2^ Estimates from a mixed-effects logistic regression model with cluster pair as the random effect and intervention nested as a random slope, adjusted for women’s age, parity, education level, and household socioeconomic score.

^3^ Estimates from a mixed-effects linear regression model with cluster pair as the random effect and intervention nested as a random slope, adjusted for women’s age, parity, education level, and household socioeconomic score.
