# Supplementary material for: Effectiveness of facility-based personalized maternal nutrition counseling in improving child growth and morbidity up to 18 months: A cluster-randomized controlled trial in rural Burkina Faso
Source: PLoS One. 2017 May 25;12(5):e0177839. doi: 10.1371/journal.pone.0177839 (PMC5444625; doi:10.1371/journal.pone.0177839)
Supplement: S1 File — (DOC) [file pone.0177839.s005.doc]

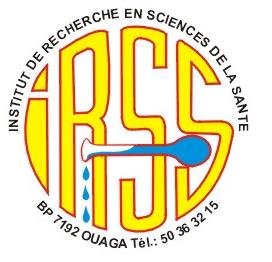

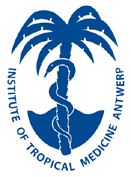


**EFFECTIVENESS OF CHILD CENTERED COUNSELING ON CHILD NUTRITION STATUS.**

**A CLUSTER RANDOMIZED TRIAL IN RURAL BURKINA FASO**.

Table des matières

[1 BACKGROUND AND RATIONALE 3](#__RefHeading___Toc455578922)

[2 OBJECTIVES 9](#__RefHeading___Toc455578923)

[3 METHODS 9](#__RefHeading___Toc455578924)

[3.1 Study area 9](#__RefHeading___Toc455578926)

[3.2 Study design 10](#__RefHeading___Toc455578927)

[3.3 The Intervention 10](#__RefHeading___Toc455578928)

[3.3.1 Improving the communicational dimension 10](#__RefHeading___Toc455578929)

[3.3.2 Improving the functional dimension 11](#__RefHeading___Toc455578930)

[3.3.3 Reorganization of health services. 11](#__RefHeading___Toc455578931)

[3.3.4 Improving the Structural dimension 18](#__RefHeading___Toc455578932)

[3.4 The intervention implementation process 23](#__RefHeading___Toc455578933)

[3.5 Population and sample size 24](#__RefHeading___Toc455578934)

[3.6 Evaluation 26](#__RefHeading___Toc455578936)

[3.7 Data collection: 28](#__RefHeading___Toc455578937)

[4 INTRODUCED CHANGES AND EXPECTED RESULTS: 29](#__RefHeading___Toc455578938)

[5 . DATA ANALYSIS: 30](#__RefHeading___Toc455578939)

[6 ETHICAL CONSIDERATIONS 30](#__RefHeading___Toc455578940)

[7 SCIENTIFIC COMMITTEE 30](#__RefHeading___Toc455578941)

[8 SUSTAINABILITY AND REPRODUCIBILITY 30](#__RefHeading___Toc455578942)

[10 REFERENCES 31](#__RefHeading___Toc455579728)

# BACKGROUND AND RATIONALE

One important target of the Millennium Development Goals (MDG) is “to halve, between 1990 and 2015, the proportion of people who suffer from hunger”. The prevalence of underweight children under-five years of age is a monitoring parameter of that target achievement . Improving child nutrition has considerable effects on children survival rates, quality of life, and individual and society development. The recent Lancet series on maternal and child under-nutrition estimated that stunting, severe wasting, and intrauterine growth restriction were together responsible for 2.2 million deaths and 21% of disability adjusted life-years (DALYs) in under-five years children . 156 million children in developing countries don’t reach their optimal development and chronic malnutrition is a major risk factor of cognitive, intellectual, and socio-emotional deficits . Under-nutrition can affect cognitive development by causing direct structural damage to the brain and by impairing infant motor development and exploratory behaviours . In the long-term, it bears under-nutrition are consequences in adult human capital and economic productivity . Moreover, better nutrition is also central for the achievement of the other MDGs, as emphasized by the SCN and the World Bank recently .

However, since the 90’s, the situation has little improved. The rate of the underweight children in developing countries decreased from 33% in 1990 to 28% in 2003. The situation is more alarming in Sub Saharan Africa, where a very slow evolution is noted . More pessimistic estimates even forecasted a rise of underweight children, from 24.0% in 1990 to 26.8% in 2015, in Africa . So that the international community recognizes today that achieving the MDGs will not be possible if the current trends (1990-2003) are maintained .

The efficacy of a number of interventions to address child under-nutrition has been thoroughly demonstrated. Those interventions include: promotion of breastfeeding (individual and group counselling); behaviour change communication for improved complementary feeding; zinc supplementation; zinc in management of diarrhoea; vitamin A fortification or supplementation; hand washing or hygienic; and treatment of severe acute malnutrition following WHO protocol . If implemented at sufficient scale, those interventions could reduce mortality between birth and 36 months by about a quarter. The development and spreading of an integrated management of childhood illnesses (IMCI) was also an important step towards better child health .

However, a major bottleneck in fighting malnutrition is precisely the incomplete and/or dysfunctional implementation of those interventions, resulting in missed opportunities of good quality management . Hindering factors vary in size, nature, and by settings, but three important dimensions can be identified:

**1. Communicational dimension:** An effective and comprehensive communication between health workers and caretakers is an essential step for an adequate management of child nutrition issues. However, health workers are often lacking that essential skill , resulting in poor efficiency of growth promotion programmes. In contrast, nutrition counselling training can change health worker behaviours and improve caregiver knowledge acquisition and child growth. However, evidence currently available has been mainly collected in urban settings with qualified health professionals. There is an obvious need of developing and evaluating the impact of such counselling training in nurses who bear the essential responsibility of providing most of the primary health care in developing country.

**2. Functional dimension:** A variety of characteristics of health service management (e.g. lack of incentives for health workers to engage in nutrition counselling; absence of monitoring of services quality; separation of curative and preventive services) hampers sustained nutrition intervention . Des-integration of IMCI is a striking example. Although part of the IMCI, nutrition is a dimension of child health often neglected by health workers . Moreover, the whole IMCI package is insufficiently implemented, most often due to the low utilization rate of health services by the population and the generally poor performance of health staff in rural settings . There is a need for innovative strategies leading to an effective implementation of all components of the IMCI in primary health care.

**3. Structural dimension:** The management of severe acute malnutrition has been well formalized in WHO protocols , and there is good evidence that where these protocols were available and sufficient attention was paid to the quality of care, case-fatality rates substantially decreased . However, the application of these protocols required numerous trained staff and a sufficient inpatient bed capacity. Moreover, in rural settings, hospital-based management results in late presentation of cases and high opportunity costs for caretakers, as well as serious risks of cross infection . Finally, the protocols are based on the utilization of treatments (F-75, F-100) which are expensive and not readily available in poor settings. Therefore, means of managing acute severe malnutrition in rural health districts where a centralized and costly, externally supported, approach is not suitable must be developed. Community-based approached are promising alternatives . However important questions arise when considering the scaling up of such program in non-emergency contexts: how can the high price of commodities are managed in the frame of limited budget allocated to health services? What is the best way to integrate programmes with health services? How can programmes be implemented with less qualified staff?

**Nutrition and health services in Houndé district.**

**Baseline assessment (April-June 2008)**

**Data collection**

**- Interviews of health workers (n=27), direct observations of the management of child illnesses (n=141) and exit interviews of caregivers (n=215) were carried out in the 27 primary health centres.**

**- Household survey (representative sample of 2164 households including 3912 children).**

**Population of under five children in the district40.000**

**Malnutrition rates in the district:**

- 16.4 % of the under-5 children are wasted and 38.7% are stunted (35.7% are underweight)
- 39% of the children ages 0-59 months had been sick during the previous two weeks

**Feeding practice**

*Breastfeeding*

-Only 47.8% of mothers have given the colostrum to their baby; 42.8% have given something to drink in the tree first days after delivery (water=64.8%). The average duration of the breastfeeding was of 15.9 ±5 months. The most frequent reason for early discontinuation of breastfeeding was: pregnancy (37.5%).

*Complementary feeding practices:*

- 39.1% of infants received complementary food before 6 months. In most of the cases the complement food given was simple millet porridge (85.5%).

-The average age to associate children to the family plate was 9.7±3.8 month.

- The average score of variety in the last 24 hours, on 9 food groups was 2.0±1.17 with a median of 2.0.

**Communicational dimension of health services**

*Curative consultation*

- 62.5% of the caregivers consulting for a sick child didn’t receive nutritional counselling; 83.0% didn’t receive the advice of increasing the fluids in case of diarrhoea; 55.3% were not advised to continue feeding the sick child.

*Reproductive health*

- 67.6% of pregnant women don’t receive food practices counselling; and 62.3% deceased her food intake in quantity, 31.2% in variety. 13.7% are declared that there is food which was forbidden them during the pregnancy. 69.8 of pregnant don’t receive any counselling on exclusive breastfeeding; 71.2% any counselling on breastfeeding practice thought the health facilities.
- Only 24.2% of interviewers had received counselling on complementary feeding though health facilities, 95.4% of them considering the advice useful

**Functional dimension of health services**

**-**The majority of facilities (85%) offer growth promotion activities during immunization sessions, but participation rates remain low (366 children in 2007).

**-** 70.6% of sick children were not weighted; 99.1% didn’t have their length/height measured; health workers failed to ask about diet modifications in 75% of the cases.

**-** There is no follow-up file for malnourished children

- 29.2% of the newborns were not weighted

**Structural dimension of health services**

-The majority of the centres (96%) have only one baby balance, but only 6 centres (22%) had length/height scale which was moreover rarely used.

-There is no functional Nutrition Rehabilitation Centre (NRC) in the district. The cases of severe malnutrition must be transferred at Bobo (more than 100km of the district hospital).

We propose to improve child nutrition in the Houndé district by an intervention which addresses those three essential dimensions of health services. During infancy and early childhood, contact with health services, particularly for curative care, is perhaps the single most common source of institutional contact between families and public health sectors services. Moreover, the credibility that health care providers are typically accorded in many social settings suggests that nutrition advice delivered by practitioners may be particularly persuasive .

First, the communicational dimension will be improved through developing a patient-centreedness approach. The patient-centreedness model developed by Stewart et al will be the guiding line of the intervention. This model takes into account six interconnecting components (figure 1): 1) Exploring both disease and illness experience, 2) understanding the whole person; 3) funding common ground regarding management; 4) incorporating prevention and health promotion; 5) enhancing the doctor-patient relationship; 6) being realistic about personal limitations and issues such as the availability of time and resources.

Second, the functional dimension will be addressed

1) by adapting the IMCI strategy to the local environment and making it the effective pivotal tool of all under-five consultations . Much has been learned from disease-specific control programs in the past 15 years. The current challenge is to apply the lessons from these programs to strategies that promote coordination and greater integration of activities in order to improve the prevention and management of childhood illness, including malnutrition. The IMCI strategy combines improved management of childhood illness with aspects of nutrition, immunization, and other important factors influencing child health, including maternal health.

2) Moreover, if one looks at the prevalence of malnutrition and the accumulation of the deficit in linear growth, it is clear that the first 18 months of life are a very vulnerable period for changes in feeding habits. Maternal nutrition during pregnancy and lactating, impacts on child growth. We know that efficient breastfeeding protects children from diseases and provides them with the most suitable food intake. Another period of risk is the weaning period where less suitable food combinations are often given to the children. In contradiction to these observations, growth promotion activities of the health services have been so far spread over the 5 first years of life, with discouraging results both in terms of follow-up rates and outcomes . We proposed to reorganize health services in order to maximize contacts between caretakers and caregivers during the critical period and support mothers in their effort to provide the best possible food to their offspring. The activities to improve child growth and development will be essentially promotional and preventive and based on regular contacts. Growth promotion activities will begin during pregnancy, and will be combined to immunization sessions and child sick consultations after birth.

Lastly, we will develop an integrated strategy of community-based rehabilitation of severe malnourished cases, and children play session to all services provided to children.

**Figure 1: The patient-centreed clinical method: six interactive components**

**2: Understanding the whole person**

Take into account the multiple aspects of the child life.

Developmental history;

Life cycle issue and feeding;

The multiple contexts in which they live including the ecosystem.

**1: Exploring child both disease and illness experience**

-Explore the caregiver feeling and idea about her child disease

-How the child illness is impacting on his functioning

What her expect from the physician

Proximal context

Distal context

Disease

Illness

History and

Physical examination

Feeling

Ideas

Function

Expectation

The children Person

**3. Funding common ground**

Defining the problem

Establishing the goals of treatment and/ or management

Identifying the roles to be assumed by the caregiver and provider

**4. Incorporating prevention and health promotion**

Counseling on feeding practice adapted to child age and illness experience,

Hygiene

Vaccination

**6. Being realistic**

About time

Improving teambuilding and teamwork

Rationalize the available resources

Mutual decision

1. **Enhancing the caregivers Provider relationship**

Each contact with the caregiver will be use to build and improve the caregiver-physician relationship by including compassion, trust, a sharing of power and healing.

Source: Moria Stewart, et al., *Patient-Centreed Medicine Transforming The Clinical Method.* PATIENT-CENTREED CARE, ed. Moria Stewart, Judith Belle Brown, and T.R. Freeman. 2003.

# OBJECTIVES

***Goals:***

To improve the nutritional status and care quality of under-5 children in Houndé district, by a three-fold intervention in primary health care services: enhancement of patient-centeredness, actual implantation of the IMCI package and community-based rehabilitation of Severe Acute Malnourished children, using local RUTF.

***Targets***

*Outcomes:*

- To reduce morbidity rate in under five children

- To reduce wasting rate in consulting children by 50% (from 16% to 8%)

- To reduce the annual incidence of severe malnutrition by 50%

- To reduce the cost of severe malnutrition care

-*Processes:*

- To improve communication and management skills of primary health workers, particularly in the domain of nutrition, by 100% (all mothers should be advised about exclusive breastfeeding and complementary feeding, instead of the current 24%; all sick children should have their weight and length/height measured at any consultation; all mothers of sick children should be asked and advised about feeding practices)

- To improve knowledge and feeding practices of caretakers by 50% (75% of the mothers will give the colostrums to the newborn; 60% of the mothers will practice exclusive breastfeeding)

- To manage cases of severe acute malnutrition in each health facilities

- To manage cases of severe acute malnutrition in line with international recommendations of good practice.

# METHODS

## Study area

The intervention will be carried out in the sanitary district of Houndé. This district counts 231.056 inhabitants with a density of 42 inhbts/km2; it includes 7 departments, 99 official villages and more than around thirty migrants' camps. On the medical level, the district counts 27 primary health centres and one hospital district. A baseline survey has demonstrated the importance of child malnutrition in the area and the current inadequacy of health services to tackle it (see box above).

## Study design

The intervention will be evaluated by a pair-matched, cluster-randomized controlled trial . Health facilities, together with the population living in their catchment area, will be matched by pair on the basis of accessibility criteria (distance to the district hospital), functioning criteria (number and profile of health workers, measles vaccine coverage, attendance rates of the growth monitoring programme, utilization rates of health facilities) and population characteristics (households socioeconomic indicators, child morbidity, rates of child wasting and stunting). Scoring of all health facilities will be based on the baseline data, and such score will be used to pair health facilities. Within each pair, one health facility will be randomly allocated the intervention, the other one serving as a comparator.

## The Intervention

### Improving the communicational dimension

A genuinecommunication between health providers and parents is an essential component for the delivery of good quality healthcare. Such communication process, based on a relationship of trust and understanding between the different actors, should address the underlying factors of the health problem and the concrete means to remedy it given the social and familial context.

To improve this aspect, all health workers in the intervention group will be trained on nutrition counselling and child–centeredness. This training will improve health provider skill in detection of feeding problems and negotiation with the mother on possible solutions that she would adopt among a locally adapted set of feeding recommendations .

The training will include two phases:

i) Academic training based on the “Manual on Counselling the Mother”, that includes specific guidance on breastfeeding and complementary feeding counselling (WHO 1997c), as well as on a specific techniques of communication involving all the aspects of the patient - centeredness concept.

ii) Practical training based on case studies in vivo.

This basic training will be reinforced by regular formative supervision sessions carried out by the district director, on a topic identified by the health workers as essential, or on weaknesses identified during the supervisions.

Health workers will be trained by two members staff of the district direction, who will be themselves trained by consultants of the Health Care Department of the Provincial Hogeschool Limburg (PHL): <http://cms.phl.be/eCache/DEF/2/129.html>.

### Improving the functional dimension

*Effective implementation of IMCI*

The health providers in the intervention group will receive training based on the Integrated Management of Childhood Illness Training Manual elaborated by the WHO and UNICEF , with a particular attention on the nutrition component. A regular supportive supervision will be introduced to accompany health workers in the Child illness assessment and care provide. Care will refer to caregiver practices and behaviours changes, Common child illness management, psychosocial stimulation and emotional support.

**Table1. Integrated care provided by first line health services**

**(Derived from WHO/CHS/CAH/98.1A REV.1 1999)**

### Reorganization of health services.

During their lifetime, children encounter a number of time periods during which they are particularly at risk of adverse health outcomes with long-lasting consequences. The intra-uterine period and the first year of life are such critical periods . However, these critical periods are not often acknowledged by health services. The Growth Monitoring and Promotion program for instance implies measuring the children monthly until age 5, i.e. at the same frequency whatsoever the actual age is. As a result, such program is inefficient and eventually not performed . Therefore we proposed to concentrate promotional and screening activities on a number of episodes during the most critical periods.

As early as the 1st antenatal visit, a systematic follow-up plan will be elaborated with the mother. The number of contacts will be standardized to nine for each child (at delivery, 6-8 weeks, 3 months, 4 months, 6 months, 9 months, 12, 15 months, 18 months). At each contact, specific services will be provided.

- **During pregnancy:**

**Table2. Health Sector and Maternal Actions to Improve Maternal Nutrition in Africa (from** )

**At delivery**:

- examine the newborn: general examination based on the IMCI framework; specific examination for sub-luxation of the hip and clubfeet, testicular descent;

- take anthropometric measurements (weight, height); give adequate recommendations as regards the home-management of low birth weight babies;

- promote and support optimal breastfeeding practice;

-promote family planning as a health and nutrition intervention;

- formulate answers to address expressed concerns of parents;

- Correct vitamin A deficiency and anemia in mother;

- vaccinate the newborn (BCG).

**At 6-8 weeks, 3months*, 4 months*, 5 months*, 9 months, 12 months* (*following the vaccination calendar)**:

-examine the infant following the IMCI framework; specific examination for hip dislocation (6-8 weeks);

-take anthropometric measurements (weight, height); discuss the growth pattern of the infant with the parents; investigate the vulnerable factors that may contribute to the potential failure to thrive in each specific case;

-promote exclusive breastfeeding; discuss concerns and difficulties related to breastfeeding and solve them; discuss feeding plans (see table 3); discuss difficulties of implementing the recommendations met by the caretakers;

- vaccinate the infant according to the national vaccination schedule;

- provide vitamin A supplements (12 months)

- ensure adequate malaria prevention (mosquito-net using), general accident prevention; discuss hygiene issues; sensitize, and encourage caretakers to consult quickly when the infant is sick.

- discuss options for family planning.

**At 15 months, 18 months:**

Two supplementary contacts to enhance the feeding practices:

-examine the infant following the IMCI framework; specific examination for hip dislocation (6-8 weeks);

-take anthropometric measurements (weight, height); discuss the growth pattern of the infant with the parents; investigate the vulnerable factors that may contribute to the potential failure to thrive in each specific case;

- discuss feeding practices; discuss difficulties of implementing the recommendations met by the caretakers;

- ensure adequate malaria prevention (mosquito-net using), general accident prevention; discuss hygiene issues; sensitize, and encourage caretakers to consult quickly when the infant is sick.

- discuss options for family planning.

**Table 3: Recommended feeding and dietary practices (derived from Linkages** )

| Infants 0 to 6 months | 􀂌 **Initiate breastfeeding within about one hour of birth.**  􀂌 **Establish good breastfeeding skills (good positioning and attachment).**  - Baby should be held close to mother, facing the breast, with the  baby’s ear, shoulder, and hip in a straight line.  - Infant’s mouth should open wide just before attaching so the  nipple, and as much of the areola as possible, are in the mouth. If  properly attached, the lips are rolled outward, with the tongue  over the lower gum.  - Signs of effective feeding include visible jaw movement drawing  milk out, rhythmical suckling with an audible swallow, and no  drawing in of cheeks.  􀂌 **Breastfeed exclusively** (no prelacteal feeds, no other foods, no  water or other liquids) for about the first six months.  􀂌 **Practice frequent, on-demand feeding, including night feeds** (8–  12 breastfeeds per 24 hours, every 2–3 hours, or more frequently if  needed).  􀂌 **In areas where vitamin A deficiency occurs, mothers should take**  **a high-dose vitamin A supplement (200,000 IU) as soon as possible**  **after delivery, but no later than eight weeks postpartum, to**  **ensure adequate vitamin A content in breastmilk.** |
| --- | --- |
| Breastfed Children 6 to 24 months | 􀂌 **Continue frequent, on-demand breastfeeding, to 24 months and**  **beyond.**  􀂌 **Introduce complementary foods beginning around six months of**  **age.**  - Breastfeed before each feeding of complementary food.  􀂌 **Increase food quantity as the child ages while maintaining frequent**  **breastfeeding.**  - Provide 6- to 8-month-old infants *approximately* 280 kcal per day  from complementary foods.  - Provide 9- to 11-month-old infants *approximately* 450 kcal per day  from complementary foods.  - Provide 12- to 24-month-old children *approximately* 750 kcal per  day from complementary foods.  􀂌 **Increase complementary feeding frequency as the child ages, using**  **a combination of meals and snacks.**  - Feed complementary foods to 6- to 8-month-old infants 2–3 times  per day.  - Feed complementary foods to 9- to 11-month-old infants 3–4 times  per day.  - Feed complementary foods to 12- to 24-month-old children 4–5  times per day.  􀂌 **Gradually increase food thickness and add variety as the child ages,**  **adapting the diet to the child’s requirements and abilities.**  - Feed mashed and semi-solid foods to infants, starting around 6  months of age.  - Feed energy-dense combinations of foods to 6- to 11-month-olds.  - Introduce “finger foods” (snacks that can be eaten by children alone)  at about 8 months of age.  - Make the transition to the family diet at about 12 months of age.  􀂌 **Diversify the diet of both the breastfeeding mother and the child by**  **including fruits, vegetables, fortified foods, and/or animal products**  **to improve quality.**  - Feed fruits and vegetables daily, especially those rich in vitamin A  and other vitamins.  - Feed meat, poultry, fish, or other animal products daily or as often as  possible (if feasible and acceptable).  - Use fortified foods, such as iodized salt, vitamin A-enriched sugar,  iron-enriched flour, or other staples, when available.  - Give vitamin-mineral supplements when animal products and/or fortified  foods are not available.  􀂌 **Practice active feeding.**  - Feed infants directly and assist older children when they feed themselves.  - Offer favorite foods and encourage children to eat when they lose interest  or have depressed appetites.  - If children refuse many foods, experiment with different food combinations,  tastes, textures, and methods for encouragement.  - Talk to children during feeding.  - Feed slowly and patiently and minimize distractions during meals.  - Do not force children to eat.  􀂌 **Practice frequent and active feeding during and after illness.**  - During illness, increase fluid intake by more frequent breastfeeding,  and patiently encourage children to eat favorite foods.  - After illness, breastfeed and give food more often than usual, and encourage  children to eat more food at each sitting.  􀂌 **Practice good hygiene and proper food handling.**  - Wash caregivers’ and children’s hands before food preparation and  eating.  - Keep all food preparation surfaces clean; use clean utensils to prepare  and serve foods.  - Cook food thoroughly.  - Avoid contact between raw foodstuffs and cooked foods.  - Serve foods immediately after preparation; avoid storing cooked  food.  - Wash fruits and vegetables.  - Use safe water.  - Use clean cups and bowls; never use feeding bottles.  - Protect foods from insects, rodents, and other animals.  - Store non-perishable foodstuffs in a safe place (separate from pesticides,  disinfecting agents, or other toxic chemicals). |

Besides the pre-established schedule of contacts, a home-based follow-up willbe made by the community health agents to families of children:

- missing two consecutive meetings of the follow-up plan

- who haven’t gained weight during the previous 3 months

Those home-visits will aim at assessing care and feeding practice, at reinforcing counselling and at indentifying rapidly a specific need of the children. Possibly, they will also create the opportunity for applying feeding demonstration. The efficacy of such home-visits has been demonstrated in a number of contexts . These community health agents are already available in the field but need to be coached and supported.

**Quality Monitoring of care provided to children and accreditation:**

As demonstrated in our baseline survey, the IMC package is currently not implemented in the health centres of the district, in spite of a previous training of the health agents. The reasons of such discrepancy will be investigated and difficulties met by health agents will be taken into account during a new training round. Quality aspects of health care will be emphasized during the training, and health agents will be accountable for the care provided. In particular, health agents will be invited to audit their practice, notably the way malnourished children have been managed. These audit sessions will be conducted by a team composed of members of the health centre, members of the community, some member of the district team and a social educator, to integrate the clinics and psycho social aspects. This team of audit will meet every two months to review the files of the malnourished children admitted meanwhile. The expected outcome of such process is a continual appraisal of the healthcare by the providers themselves in order to identify and implement possible improvements. Audit reports will be used to orientate supportive supervision and will be one element to deliver the accreditation. Every year health facilities successful in reorganizing their services and fulfilling quality criteria will be delivered a label of “Child Friendly Health Centre”. Such accreditation scheme has contributed to stimulating services upgrade in other settings.

A committee of accreditation composed of a representative of every health centre’s management committee, two representatives of the district team, two representative of the regional direction of the health will be put in place at the beginning of the project.

The following criteria will be used for the range of the centres and will be refined by the committee of accreditation: The rate of frequentation of the health centre, the rate of completive of the follow up plan, the rate of abandonment... The health workers particularly devoted will receive special prices every year. The financing of these prices will be assumed by the different management committee and supported by the project.

**Enhancing the interface between the caretakers and the healthcare providers**

The existing community health workers will be trained to child nutrition, adequate feeding practices, to the key period of the child's life and the specific needs at every period.

They will play and accompaniment role of families with under five children. Each community health worker will be affected to a number of families, and have to:

- follow these families at home and incite them to use the health centre in the setting of the child's systematic follow-up plan and period of disease,
- look for the children who are not regular to the follow-up plan, and identify possible problems and to encourage them to use the health centre,
- Identify cases of home delivery and encourage those families to bring the newborn in the health centre for the follow-up.
- accompany health workers in the home visits for malnourished children
- Assess the nutritional status by measuring the mid-upper arm circumference (MUAC) and refer the children with a MUAC<12, 5 cm to the health centre for further assessment. The purpose is to detect quickly moderate acute malnutrition case for a community ambulatory care.

### Improving the Structural dimension

To date, no structure is in place to manage cases of severe malnutrition. The intervention at this level will be twofold. First, to ensure that cases of severe malnutrition are appropriately managed at the reference level and treated there until they recover to the level of moderate malnutrition; second that cases of moderate malnutrition are adequately managed at the health centre level. The main aims are: to provide effective care for acute malnutrition, by using as much as possible local resources; to improve access and acceptability of the programme for the population; to reduce length of hospitalisation in the health centre, diminishing cross infection.

**Upgrading the reference hospital paediatric services**

It has been proposed that the WHO guidelines for severe malnutrition are feasible in rural African hospitals, but at the condition of implementing other interventions that build human capital and strengthen the health system .

In order to adequately manage cases of severe malnutrition at the district hospital as recommended by the WHO, the following actions will be undertaken:

- Case detection in the primary health facilities,

- Reference in the district hospital,

- Inpatient care and management of complications in the district hospital,

- Contre-reference in the primary health care as soon as the complication are treated,

- Regular supportive supervision of the primary health care by the district hospital team

- Clinical audit of all case admitted in the district hospital.

Cases of severe malnutrition and cases of moderate malnutrition with complications will enter in program with two phases (the inpatient program and the outpatient program).. Severe malnutrition is defined as W/H<70% or presence of bilateral oedema. Complications are defined as one of the following conditions: anorexia, acute respiratory infection, severe dehydration, severe anaemia, apathy.

**The inpatient program in the district hospital paediatric services**

The treatment will be essentially based on the OMS standard protocols . However, the WHO guidelines recommend a W/H>90% as criterion of a successful treatment. To reach such a target takes several weeks, exposing the children to possible cross-infections in the hospital and increasing the burden of the parents. Within the current intervention, children will be referred to the primary health centre level for an ambulatory follow-up as soon as the complications are treated and the WH>70%, i.e. when the child is not anymore severely malnourished although not yet completely cured (see point 3.3).

a) Inpatient-treatment phase 1 using only F75 Formula

- Admitted children should be registered and all information recorded in a [Multichart](javascript:plainWindow1()) including the target weight for discharge (WHO/NCHS table).

-Admitted children should be provided with a systematic medical examination and given routine medicine.

- Only F75 will be use during this phase

- Daily surveillance of weight will measured and plotted on the multichart.

- The degree of oedema will assessed and noted in the Multichart,

- Body temperature will measured twice a day

- Standard clinical signs (stool, vomiting, dehydratation, cough, respiration and liver size) will assessed

- Breastfeeding children should always get the breast milk before the diet and on demand.
- Preparation of F75 to give during this phase will based on the class of weight

-The WHO Feeding Table will be used

b) Inpatient-treatment transition using only RUTF

- Daily surveillance of the child remains exactly the same in Transition phase as it was in Phase 1. The expected rate of weight gain is about 6g/kg/day if all the food is taken by the children and there is not excessive malabsorption.

- Breastfeeding children should always get the breast milk before the diet and on demand.
 - Routine antibiotics should be continued after transferred from Phase 1 for another four days.

**Move the children back to phase 1:**

- If the child gains weight more rapidly than 10g/kg/day
- If there is increasing oedema
- If child suddenly develops oedema
- If liver size increases rapidly
- If child develops signs of fluid overload
- If child develop signs of abdominal distension
- If child gets significant re-feeding diarrhoea so there is weight loss
- If naso-gastric tube is needed
- If complication arise that necessitates an intravenous infusion

***Progress the child to phase 2***

- If the child has a good appetite. Taking 90% of the prescribed RUTF
- If the child has lost the oedema entirely

**Phase 2 outpatient treatments in the primary health center**

All children with severe acute malnutrition without medical complications and that have passed the [appetite test](http://motherchildnutrition.org/malnutrition-management/info/appetite-test.html) can go directly to Phase 2.

Children that are admitted directly to Phase 2 as out-patients should be provided with a systematic medical examination and given routine medicine.

Children transferred from [in-patient treatment](http://motherchildnutrition.org/malnutrition-management/management-severe-acute-malnutrition/in-patient-treatment-phase-2.html) should be registered as "transferred from".

Surveillance of the children:

- Every week, weight will measure, entered and plotted on the Child Card.
- Every week, the presence of bilateral [oedema](http://motherchildnutrition.org/early-malnutrition-detection/detection-referral-children-with-acute-malnutrition/screening-for-acute-malnutrition.html#Bilateral%20pitting%20oedema) will assess and noted in the Child Card.
- Every week, body temperature is measured and noted in the Child Card.
- Every week, standard clinical signs (stool, vomiting, dehydration, cough, respiration and liver size) will assessed and noted in the Child Card.
- Every week, [Mid-Upper Arm Circumference (MUAC)](http://motherchildnutrition.org/early-malnutrition-detection/detection-referral-children-with-acute-malnutrition/screening-for-acute-malnutrition.html" \l "Mid-upper Arm Circumference (MUAC)) will take.
- Every month measurement of height/length

**RUTF** is provided as take-home therapeutic food for malnourished children only.

- The amount of RUTF provided to the caregiver will base on the class of weight and on the necessary quantity required to last until the next visit to the out-patient site.
- Use the RUTF Feeding Table
- Breastfeeding children will always give Breast-Milk before RUTF.
- Move the child in phase 1 (in-patient)
- If the child develops any medical complications that demand in-patient treatment
- If the child has severely reduced appetite
- If the child increases/develops oedema
- If the child develops “refeeding diarrhoea” sufficient to lead to weight loss,
- If the child does not respond to the treatment
- If there is a weight loss for 2 consecutive weighing sessions
- If there is a weight loss of more than 5% of body weight at any visit,
- If the weight stays for three consecutive weighing sessions.

**Ensuring ambulatory care for moderately malnourished children**

All case of moderate acute malnutrition (70 %< W/H <80%) will enter in an ambulatory program emphasizing an appropriate child-centered counselling on feeding practice and care. Home visits will be made once a week to assess feeding practices and child specifics problems, and needs. Although following the main recommendations reported in table 3, the rehabilitation will be thus tailored to each case. Two important aspects will be stressed: frequency and energy density of meals. Means to improve these two aspects will be defined in accounting for the purchase power and the food accessibility of each family. Families will be encouraged to use local foods, e.g. add 5 ml of oil in the gruel ration and provide the child with snacks several times during the day. RTUF could be purchased at the health centre. The follow-up of the nutritional status will be made once a week in the health centre. During every visit the anthropometric measures will be taken (weight height, MUAC) to follow the evolution and the presence of other diseases will be assessed for providing adequate treatment.

A child will be confirmed cured when his/her W/H>80% and growth curve ascending for two consecutive weighing (2 consecutive weeks).

A particular attention will be paid to children not responding to the programme in order to adapt rapidly and adequately the management. Children who have not gained weight after two weeks of follow-up will be thoroughly examined to assess the presence of chronic pathologies (cardiac malformation, renal deficiency, HIV infection, pulmonary tuberculosis) and begin an adequate treatment, if possible. If a chronic pathology is ruled out, the family will be encouraged to use RTUF. Children who developed a medical complication and/or loose weight will be transferred to the hospital.

**Admission criteria:**

**Children from 6 to 59 months for which the P/T<70% or MUAC <110 mm**

**Check for complications and do the appetites test**

**Children with complication and/or failing appetite test.**

1. Bilateral pitting oedema grade 3*(severe oedema)

OR

2. MUAC<110 mm AND bilateral pitting oedema grades 1 or 2 (marasmic kwashiorkor)

OR

3. MUAC<125 mm OR bilateral pitting oedema grades 1 or 2

AND one of the following:

Anorexia

Lower respiratory tract infection

Severe palmer pallor

High fever

Severe dehydration

Fails appetite

**Children with appetite, and no complication.**

1. MUAC<110 mm

OR

2. Bilateral pitting oedema grades 1 or 2 with MUAC>110 mm

AND

Appetite

Clinically well

**Phase 2 Out-patient therapeutic care**

**RUTF follow-up in the primary health facilities**

**Fails appetite or develops medical complication.**

Phase 1: inpatient care in the district hospital

IMCI / WHO protocols

**Return of appetite and reduction of oedema**

Transition phase

In-patient with RUTF

Discharge to follow up

Schema for the decision making process

## The intervention implementation process

The intervention will be integrated to usual activities addressing to young children in the health facilities. Delivery of the intervention will be monitor, by the local health team at routine monthly review meetings. The investigators role will be restricted to the measurement of outcomes.

* At the national and regional level:

A workshop will be organized by the project team to present the project, to the head of the ministry of health and his partners (UNICEF, WHO NGO…). The main objective is to obtain their adhesion and accompaniment, and the utilization of the result in the national level.

* On the level of the medical district of Houndé: A local workshop will be organized by the project team; during which the project will be presented. Will be invited to this workshop: the district team, the local authority and health centre committee management, the social actors, the communities’ heads, the local NGO and women associations, the partner of development… The objective is to obtain their adhesion and their accompaniment.

* On the level of the primary health centers: four strategies of installation are considered:

1) *Information of the health workers on the result of the baseline, the extent and the importance of* the nutritional problems, the effects of malnutrition on children health, their growth and their development.

*2) A basic training on IMCI modules,* the nutritional needs for pre-school children, to *the nutrition counseling*, to the interpersonal communication, in the patient centered approach, to a better control of the techniques of weighing and measurement of the children, the prescription and the regulation of the RUTF.

*3)*  Setting up a nutrition team in each health centre composed of the community health workers and the play leaders and two health workers. This team will undertake the execution of the activities envisaged in the protocol and the adaptation of this one to the local context, by taking account of opportunities and obstacles present. The methodology of work of this group will consist in following the cycle of research action , which is in fact a process of continuous training. Indeed, the methodology of research action answers well the need for continuous adaptation of the theoretical and scientific bases to the real context, and takes account of the importance to develop the autonomy of the health professionals in their capacity of adaptation.

4) Setting up of the accreditation committee and the audit team.

## Population and sample size

This study concerns all the health workers in the primary health facilities of the Houndé district and the children from 0 to 24 months as well as their mother or caretakers. The intervention unit is the health center and the population living in its catchment area, i.e. health centers will be randomized.

For the evaluation as well of the efficiency as of the impact of this intervention, the statistical unit will be consisted the center of health and its population of cover. On the level of each statistical unit, three types of samples will be necessary in comparison with the study populations:

- Health workers

A random sample of one health worker will be made by center.

*Inclusion criteria*: Personnel of health having at least one year of activity in the center.

Caregivers

Insofar as several outcomes used to compare the two groups of mother, the principal outcome used to compare the two groups, will be the proportion of caregivers having a good score of Knowledge on adequate breastfeeding and complementary feeding practices.

Several hypothesis have been considered to estimate the sample size. The reference method for the sample size calculation is the paper of Hayes et al for cluster design; we used equation N°4 of this paper

C=number of pairs=6 in our case Km = intracluster correlation coefficient; in the case of paired groups, 0, 25 is used as conservatory value.

= proportion of caregivers with a good score of Knowledge in the control group

= proportion of caregivers with a good score of Knowledge in the intervention group

n=sample size by cluster

The value of C being known in our case, we deduced from this equation, the values of n, according to several assumptions on the rate of caregivers with good knowledge and practices on breastfeeding and complementary feeding and according to two levels of power 1-β=0,80 or 0,90. This is shown in the table below.

**Estimate of the number of caregivers** necessary to compare the two groups

| = 0,25 | α=0,05  1-β=0,80 | α=0,05  1-β=0,90 |
| --- | --- | --- |
|  | n=15 | n=50 |
|  | n=33 | n=95 |
|  | n=11 | 20 |

By taking a maximum power, a sample of 95 women per cluster will be necessary, which is 1140 women in total.

*Inclusion criteria*: all women of childbearing age having a child into care from 0 to 59 months and living in the health center catchment area for at least one year at the time of the survey.

Children from 0 to 24 months

Taking into account the duration of the intervention a notable change on the child nutritional status could be measured at the level of wasting; which is relevant to measure in children 0 to 24 months. The primary outcome, to compare the two groups will be the proportion of wasted children that is to say the proportion of children with weight for height Z score ≤ -2. According to the baseline data, this proportion was 16.4 on average in the district. The same equation cited above was used to estimate the number of required children. We considered the hypothesis that, after the intervention, the proportion of wasted children would decrease from 16.4% to 10% in the intervention area. Considering two levels of power the numbers of required children is summarized in the table below.

Table IV: Estimate of the number of children necessary to compare the two groups

| = 0,25 | α=0,05  1-β=0,80 | α=0,05  1-β=0,90 |
| --- | --- | --- |
|  | n=180 | n=650 |

We also considered other values of:

| = 0,20 | α=0,05  1-β=0,80 | α=0,05  1-β=0,90 |
| --- | --- | --- |
|  | n=115 | n=200 |

| = 0,30 | α=0,05  1-β=0,80 | α=0,05  1-β=0,90 |
| --- | --- | --- |
|  | n=800 | n=5000 |

We finally kept our first hypothesis with = 0.25, 1-β = 0.80 which gives n = 180 (180 children per cluster).

I*nclusion criteria*: all children 0 to 24 months whose parents residing in the health center catchment area for at least one year at the time of the survey.

The selection of women and children will be in population and randomly. In contrast, the number of children required (180 by cluster) being greater than the number of women (95 per cluster), and to be more operational, we will choose to include in the study women of childbearing age with at least a child under 24 months. This will allow us to select our two populations at the same time.

## Evaluation

The evaluation will based on the program **theory**, both processes and outcomes being assessed (see figure 3).

**The intervention process** will be assessed in all the 12 health facilities using structured observations of health facilities activities, regular meeting in the health facilities, interview of caregiver and health workers, and a cross-sectional survey.

**Figure 3:** The programme theory

**Inputs**

Staff

Equipment

**Supportive supervision**

**Improving functioning**

-Training Health care providers in IMCI

- Reorganizing GMP activities

- Introducing home visits for sick and severe malnourished children

-Introducing an integrated accreditation scheme for health facilities

**Improving structure**

- Production of a Ready to Use Therapeutic Food

- Adapt management of severe malnutrition cases to local conditions

- Integrated psychosocial stimulation

**Outputs**

Health workers communication and management skills in nutrition improved

Health providers knowledge about nutrition counseling and practices improved

Caretakers knowledge about basic nutrition counseling and complementary feeding practices improved

Caretakers satisfaction with health care, caretakers compliance with recommended feeding practices

Effectiveness of acute malnutrition case management

Effectiveness of psychosocial stimulation activities

**Outcomes to measure**

Child morbidity and mortaliry improved

Child dietary intake improved

Improved children nutritional status

Improved child psychomotor development.

**Improving communication**

-Training healthcare providers in patient-centreedness and communication

**The intervention component**

**The intervention impact on health facilities functioning, child health and nutrition status:**

*Primary outcomes:*

- Child weight and height at 6 and 12 months

-rate of acute malnutrition

*Secondary outcomes*:

-Health provider’s knowledge about nutrition counseling and practices

-Caretakers knowledge about basic nutrition counseling, and breastfeeding and complementary feeding practices

- Caretakers satisfaction with health care

-Implementation of recommended feeding practices

-Child dietary intake

**Evaluation tools:**

The reference guidelines of the IMCI program evaluation and the modules of assessment of the quality of the nutrition cares developed by HKI will be adapted, and will be used for the assessment of the different components of the intervention .

## Data collection:

Data will be collected by trained field workers who are not involved in the delivery of the intervention:

-Interview through a structured questionnaire before, 3 month after the training of health workers and at the end of the intervention to test their knowledge on child nutrition counselling;

- Direct observation of five sick children consultation, and five preventive consultation with each provider, at the same time (before training, 3 month and 12 after the training) to test health providers’ skills in child health and nutrition management, their practice of communication and attitudes with regard to the children and their parents, the aspects related to the context of the meeting (latency, duration of the consultation…) and the quality of care provided.

- Exit interview with caregivers to measure their satisfaction as regards the services offered (growth promotion Program, curative care, communication and relation between health workers, nutrition counseling, malnutrition cases management), their practical knowledge, aptitudes as regards pregnant woman and the young child feeding practices, adapted care, family planning, etc

- At 3, 9 and 12 months, morbidity, health facilities use, anthropometry assessment, breastfeeding/complementary feeding practices reported on at 24-hour dietary recall and child psychosocial development assessment will be made.

# INTRODUCED CHANGES AND EXPECTED RESULTS:

**The principal changes introduced** will be in the structural dimension of health system:

- The decentralization of severe acute malnutrition case management in the primary health centres,

- The production and introduction of a local Ready to Use Therapeutic Food in the health system.

- At Community level, the families will be indirectly concerned in the detection and the rehabilitation of malnutrition case.

**The expected effects of this intervention are at two levels**:

*1°) On the level of the outputs*: health providers should increase their knowledge on child health and nutrition (knowledge). They should have ability to evaluate child nutritional state, to analyze the causes and to identify the adequate initiatives. They should be more effective in the child growth promotion activities, child illness and malnutrition management. The providers will enhance their skills on communication and nutrition counseling with caregivers; they will be more motivated.

Caretakers will find more satisfaction with health care (sympathy and answers adapted to their waiting). This should lead to an increase in the use of the curative and preventive services, a greater regularity and a larger cover of the services, a better tracking of the cases of malnutrition, a reduction severe acute malnutrition cases, good practices in pregnant woman and young child feeding (suitable (breast feeding, weaning…). For the malnourish children, fast rehabilitation is awaited, at a low cost, with a reduced probability of decline on the level of the family.

2) *On the level of the outcomes*:

More frequent and more appropriate use of health services, and changes in food practices, an improvement of the child development, nutritional and medical state is awaited. This one will be measurable in terms of vaccine cover, improved nutritional indicators and by a reduced morbidity.

# . DATA ANALYSIS:

A double entry by two distinct operators, will be done using the software EPIDATA . The comparison of the two data bases will make it possible to correct the major errors.

After cleaning, a scoring will be established for the various indicators. The quantitative variables will be categorized in discrete variables, some synthetic scores will be built for the variables expressing same dimension, and the analysis will be done on software STATA version 10 in three stages. Linear regression and logistic regression will be used to assess the effect of the intervention on continuous and dichotomic variables, respectively. Mixed models will be used for all analysis to account for the cluster randomization and the repeated measurements at the individual level. Qualitative data will be coded and analyzed in QSR 8.0

# ETHICAL CONSIDERATIONS

The consent

All participants in the study will be informed of the study aims and methods, and will be invited to provide a written or oral informed consent.

Approval of the Ethic committee

The protocol was examined and approved by the ethical committee of health research of the ministry of health of Burkina Faso, and the local health authorities.

# SCIENTIFIC COMMITTEE

A scientific committee composed by a senior in public health nutrition, and Persons in charge for the National Management of the Nutrition of the Ministry of Health of Burkina Faso. The principal researcher will be charged to establish these contacts as of the starting of the preparatory phase. The goal is to inform all the people concerned on progress and the results of research with the aim of develop the policies of health concerning the pre-school children.

# SUSTAINABILITY AND REPRODUCIBILITY

The intervention will be integrated in the IMCI program of health services. The district staff was associated in the protocol design, and will be the means actor of the intervention implementation.

# REFERENCES

1. Engle, P.L., et al., *Strategies to avoid the loss of developmental potential in more than 200 million children in the developing world.* Lancet, 2007. **369**(9557): p. 229-42.

2. Black, R.E., et al., *Maternal and child undernutrition: global and regional exposures and health consequences.* Lancet, 2008. **371**(9608): p. 243-60.

3. Grantham-McGregor, S., et al., *Developmental potential in the first 5 years for children in developing countries.* Lancet, 2007. **369**(9555): p. 60-70.

4. Walker, S.P., et al., *Child development: risk factors for adverse outcomes in developing countries.* Lancet, 2007. **369**(9556): p. 145-57.

5. Levitsky, D.A. and B.J. Strupp, *Malnutrition and the brain: changing concepts, changing concerns.* J Nutr, 1995. **125**(8 Suppl): p. 2212S-2220S.

6. Upadhyay, S.K., K.N. Agarwal, and D.K. Agarwal, *Influence of malnutrition on social maturity, visual motor coordination & memory in rural school children.* Indian J Med Res, 1989. **90**: p. 320-7.

7. Mendez, M.A. and L.S. Adair, *Severity and timing of stunting in the first two years of life affect performance on cognitive tests in late childhood.* J Nutr, 1999. **129**(8): p. 1555-62.

8. Kar, B.R., S.L. Rao, and B.A. Chandramouli, *Cognitive development in children with chronic protein energy malnutrition.* Behav Brain Funct, 2008. **4**: p. 31.

9. Victora, C.G., et al., *Maternal and child undernutrition: consequences for adult health and human capital.* Lancet, 2008. **371**(9609): p. 340-57.

10. United Nation System Standing Committee on Nutrition, *Report of the Standing Committee on Nutrition at its Thirty-first Session.* 2004, United Nation System: New York. p. 31.

11. World Bank, et al., *Repositioning Nutrition as Central to Development: A Strategy for Large Scale action (Directions in Development).*

. The International BanK for Reconstruction and Development. 2006, Washington: World Bank.

12. Fond des Nations Unies pour l'enfance, *Progrès pour les enfants : Un bilan de la nutrition*, in *UNICEF*. 2006: New York.

13. de Onis, M., et al., *Estimates of global prevalence of childhood underweight in 1990 and 2015.* Jama, 2004. **291**(21): p. 2600-6.

14. Organisation Mondiale de la Santé, *La santé et les Objectifs du Millénaire pour le Développemnt*, in *OMD, Santé et politique de développement*. 2005, OMS: Genève p. 84.

15. Sachs, J.D. and J.W. McArthur, *The Millennium Project: a plan for meeting the Millennium Development Goals.* Lancet, 2005. **365**(9456): p. 347-53.

16. Bhutta, Z.A., et al., *What works? Interventions for maternal and child undernutrition and survival.* Lancet, 2008. **371**(9610): p. 417-40.

17. Dewey, K.G. and S. Adu-Afarwuah, *Systematic review of the efficacy and effectiveness of complementary feeding interventions in developing countries.* Matern Child Nutr, 2008. **4 Suppl 1**: p. 24-85.

18. Ashworth, A., R. Shrimpton, and K. Jamil, *Growth monitoring and promotion: review of evidence of impact.* Matern Child Nutr, 2008. **4 Suppl 1**: p. 86-117.

19. Patwari, A.K. and N. Raina, *Integrated Management of Childhood Illness (IMCI): a robust strategy.* Indian J Pediatr, 2002. **69**(1): p. 41-8.

20. Bryce, J., et al., *Programmatic pathways to child survival: results of a multi-country evaluation of Integrated Management of Childhood Illness.* Health Policy Plan, 2005. **20 Suppl 1**: p. i5-i17.

21. Hampshire, R.D., et al., *Delivery of nutrition services in health systems in sub-Saharan Africa: opportunities in Burkina Faso, Mozambique and Niger.* Public Health Nutr, 2004. **7**(8): p. 1047-53.

22. Chopra, M., et al., *Effect of an IMCI intervention on quality of care across four districts in Cape Town, South Africa.* Arch Dis Child, 2005. **90**(4): p. 397-401.

23. Roberfroid, D., et al., *Perceptions of growth monitoring and promotion among an international panel of district medical officers.* J Health Popul Nutr, 2005. **23**(3): p. 207-14.

24. Roberfroid, D., G.H. Pelto, and P. Kolsteren, *Plot and see! Maternal comprehension of growth charts worldwide.* Trop Med Int Health, 2007. **12**(9): p. 1074-86.

25. Penny, M.E., et al., *Effectiveness of an educational intervention delivered through the health services to improve nutrition in young children: a cluster-randomised controlled trial.* Lancet, 2005. **365**(9474): p. 1863-72.

26. Zaman, S., R.N. Ashraf, and J. Martines, *Training in complementary feeding counselling of healthcare workers and its influence on maternal behaviours and child growth: a cluster-randomized controlled trial in Lahore, Pakistan.* J Health Popul Nutr, 2008. **26**(2): p. 210-22.

27. Santos, I., et al., *Nutrition counseling increases weight gain among Brazilian children.* J Nutr, 2001. **131**(11): p. 2866-73.

28. Pelto, G.H., et al., *Nutrition counseling training changes physician behavior and improves caregiver knowledge acquisition.* J Nutr, 2004. **134**(2): p. 357-62.

29. Tawfik, Y.M., S. Legros, and C. Geslin, *Evaluating Niger's experience in strengthening supervision, improving availability of child survival drugs through cost recovery, and initiating training for Integrated Management of Childhood Illness (IMCI).* BMC Int Health Hum Rights, 2001. **1**(1): p. 1.

30. Bodart, C., et al., *The influence of health sector reform and external assistance in Burkina Faso.* Health Policy Plan, 2001. **16**(1): p. 74-86.

31. WHO, *Management of severe malnutrition: a manual for physicians and other senior health workers.* . 1999, WHO: Geneva.

32. WHO., *Improving child health—IMCI: the integrated approach.* 1997, World Health Organization: Geneva.

33. WHO, *Management of the child with a serious infection or severe malnutrition : guidelines for care at the fi rst-referral level in developing countries.* 2000, World Health Organization: Geneva.

34. Ahmed, T., et al., *Mortality in severely malnourished children with diarrhoea and use of a standardised management protocol.* Lancet, 1999. **353**(9168): p. 1919-22.

35. Khanum, S., A. Ashworth, and S.R. Huttly, *Controlled trial of three approaches to the treatment of severe malnutrition.* Lancet, 1994. **344**(8939-8940): p. 1728-32.

36. Deen, J.L., et al., *Implementation of WHO guidelines on management of severe malnutrition in hospitals in Africa.* Bull World Health Organ, 2003. **81**(4): p. 237-43.

37. Ashworth, A., et al., *WHO guidelines for management of severe malnutrition in rural South African hospitals: effect on case fatality and the influence of operational factors.* Lancet, 2004. **363**(9415): p. 1110-5.

38. Collins, S., et al., *Management of severe acute malnutrition in children.* Lancet, 2006. **368**(9551): p. 1992-2000.

39. Moria Stewart, et al., *Patient-Centered Medicine Transforming The Clinical Method.* PATIENT-CENTERED CARE, ed. Moria Stewart, Judith Belle Brown, and T.R. Freeman. 2003.

40. WHO and UNICEF., *HANDBOOK: Integrated Management of Childhood Illness*

2005: Department of Child and Adolescent Health and Development (CAH). World Health Organization.

41. Ashworth, A., *Efficacy and effectiveness of community-based treatment of severe malnutrition.* Food Nutr Bull, 2006. **27**(3 Suppl): p. S24-48.

42. Collins, S., *Treating severe acute malnutrition seriously.* Arch Dis Child, 2007. **92**(5): p. 453-61.

43. Campbell, M.J., A. Donner, and N. Klar, *Developments in cluster randomized trials and Statistics in Medicine.* Stat Med, 2007. **26**(1): p. 2-19.

44. World Health Organiation, *Integrated Management of Childhood Illness: Adaptation Guide. Working Draft, Document ref WHO/CHD/97-3E Version 3.*

. Division of Child Health and Developement . . 1997, Geneva, Switzerland.: WHO.

45. WHO and UNICEF., *Infant and Young Child Feeding Counseling: An Integrated Course.* World Health Organization 2006.

46. Lumey, L.H., *Decreased birthweights in infants after maternal in utero exposure to the Dutch famine of 1944-1945.* Paediatr Perinat Epidemiol, 1992. **6**(2): p. 240-53.

47. Osmond, C., et al., *Early growth and death from cardiovascular disease in women.* Bmj, 1993. **307**(6918): p. 1519-24.

48. Roberfroid, D., et al., *Do growth monitoring and promotion programs answer the performance criteria of a screening program? A critical analysis based on a systematic review.* Trop Med Int Health, 2005. **10**(11): p. 1121-33.

49. The Regional Centre for Quality of Health Care at Makerere University in Uganda, LINKAGES, and USAID, *Essential Health Sector Actions to Improve Maternal Nutrition in Africa.* 2001.

50. Karaolis, N., et al., *WHO guidelines for severe malnutrition: are they feasible in rural African hospitals?* Arch Dis Child, 2007. **92**(3): p. 198-204.

51. Grodos, D. and Mercenier P, *Health systems research: a clearer methodology for more effective action.* ITG Press, 2000. **15**: p. 1-106

52. Hayes, R.J. and S. Bennett, *Simple sample size calculation for cluster-randomized trials.* Int J Epidemiol, 1999. **28**(2): p. 319-26.

53. Institut National de la Statistique et de la Démographie and Macro International Inc., *Enquête démographique et de santé, Burkina Faso 2003-2004*. 2004, Macro International Inc.: Claverton, Maryland. p. 248.

54. Helen Keller International, *Evaluation qualitative des prestations de nutrition.*, ed. division d’Helen Keller Worldwide HKI.

55. Lauritsen J BM, *EpiData (version 3)*, in

(2003-3004), The EpiData Association,: Odense, Denmark.
